# Foudroyant cerebral venous (sinus) thrombosis triggered through CLEC-2 and GPIIb/IIIa dependent platelet activation

**DOI:** 10.1038/s44161-021-00017-1

**Published:** 2022-02-10

**Authors:** David Stegner, Vanessa Göb, Viola Krenzlin, Sarah Beck, Katherina Hemmen, Michael K. Schuhmann, Barbara F. Schörg, Christian Hackenbroch, Frauke May, Philipp Burkard, Jürgen Pinnecker, Alma Zernecke, Peter Rosenberger, Andreas Greinacher, Bernd J. Pichler, Katrin G. Heinze, Guido Stoll, Bernhard Nieswandt

**Affiliations:** 1 Institute of Experimental Biomedicine, University Hospital, University of Würzburg, Würzburg, Germany; 2 Rudolf Virchow Center, University of Würzburg, Würzburg, Germany; 3 Department of Neurology, University Hospital Würzburg, Würzburg, Germany; 4 Werner Siemens Imaging Center, Department of Preclinical Imaging and Radiopharmacy, Eberhard Karls University of Tübingen, Tübingen, Germany; 5 Department of Anesthesiology and Intensive Care Medicine, University Hospital, Tübingen, Germany; 6 Institute of Immunology and Transfusion Medicine, University Medicine Greifswald, Greifswald, Germany; 7 Cluster of Excellence iFIT (EXC 2180) ‘Image-Guided and Functionally Instructed Tumor Therapies’, Eberhard Karls University Tübingen, Tübingen, Germany; 8 Present Address: Center for Thrombosis and Hemostasis, Johannes Gutenberg University Medical Center, Mainz, Germany; 9 Present Address: CSL Behring Innovation GmbH, Marburg, Germany

**Keywords:** Stroke, Preclinical research, Platelets

## Abstract

Cerebral venous (sinus) thrombosis (CVT) is an unusual manifestation of venous thrombosis causing severe neurological impairment and seizures^1,2^. Molecular mechanisms underlying CVT, potentially involving pathological platelet activation, are unknown. Here we show that antibody-(INU1-fab)-induced cooperative signaling of two platelet receptors, C-type lectin-like receptor-2 (CLEC-2) and GPIIb/IIIa, triggers within minutes a CVT-like thrombotic syndrome in mice, characterized by tonic–myoclonic seizures, platelet consumption and death. Brain autopsy showed thrombi mainly in the cortical venules, but no intracranial hemorrhages or edema formation. Transcranial intravital microscopy revealed rapidly progressing thrombosis in the superior sagittal sinus, a main site of CVT in humans. Interfering with CLEC-2 signaling or inhibition of GPIIb/IIIa completely blocked platelet activation and CVT. Blocking GPIIb/IIIa after onset of neurological symptoms protected mice from platelet consumption, CVT and death, which was not seen after treatment with heparin. These results point to aberrant platelet activation as a major trigger of CVT and potential target for treatment.

## Abstract (web summary)

Cerebral venous thrombosis (CVT) is a rare life-threatening disease of unknown etiology. Stegner et al. show that aberrant platelet activation through cooperative signaling of CLEC-2 and GPIIb/IIIa receptors triggers foudroyant CVT in mice and shed light on the molecular pathways of the disease pathogenesis.

## Main

Thrombosis, which can occur in both arteries and veins, is the leading cause of death worldwide^3^. While arterial thrombosis mainly follows atherosclerotic plaque rupture^4^, venous thrombosis (VT) typically occurs without endothelial injury at sites of blood stasis or in response to immunological stimuli, the latter referred to as ‘immunothrombosis’^5^. The cerebral venous system, comprising the cerebral veins and the dural venous sinuses, is an unusual site of thrombosis. CVT mainly occurs idiopathically, but young women are at an increased risk as a result of oral contraceptive use, pregnancy and puerperium^2^. Cancer and infections are other confounders, but a mechanistic explanation for the predilection for the brain vasculature is lacking^2^.

Thrombus formation encompasses a complex interplay of platelets and the coagulation system. Initial platelet tethering is mediated by glycoprotein (GP)Ib, followed by cellular activation, degranulation, and the functional upregulation of adhesion receptors, notably the fibrinogen receptor GPIIb/IIIa, which mediates platelet aggregation. Platelet activation is driven mainly by two major classes of membrane receptors. While locally produced/released soluble agonists (for example, thrombin, ADP) activate G protein-coupled receptors^6^, macromolecular, mostly immobilized ligands activate immune-like receptors that signal through an immunoreceptor tyrosine-based activation motif (ITAM). Among them, the collagen receptor GPVI^7^ and CLEC-2 have been implicated in injury-related thrombus formation^8,9^. The only established CLEC-2 ligand is podoplanin^10^, a transmembrane protein expressed outside the vasculature, including lymphatic endothelial cells, lung epithelial cells and the brain^11^. Podoplanin is upregulated in many cancers and in different immune cells during inflammation^12^, indicating a possible role in immunothrombosis^13^.

## Monovalent targeting of CLEC-2 triggers CVT in mice

**Fig. 1:**
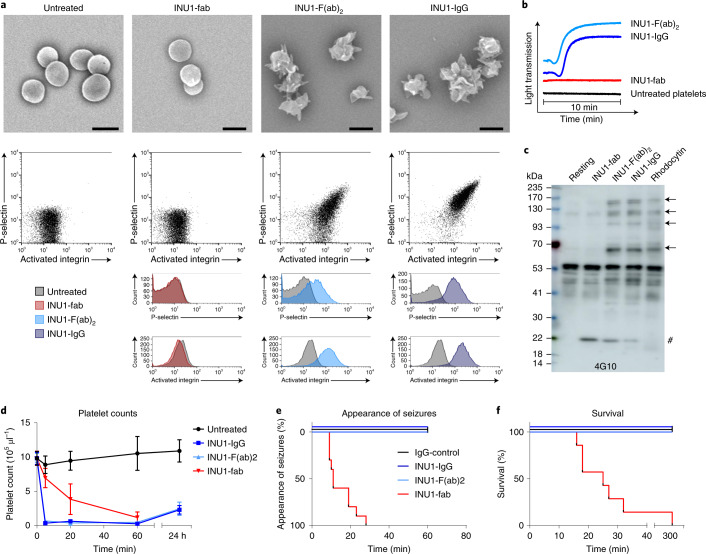
CLEC-2 dimerization, but not binding of INU1-fab triggers platelet activation. **a**, SEM (upper panels, scale 2 µm) and flow cytometry (lower panels) reveal platelet activation on binding of a divalent agent (INU1-IgG or INU1-F(ab)_2_), but not in response to INU1-fab (all antibody derivatives were added at 10 µg ml^−1^ final concentration [f.c.]) in vitro. Representative images of *n* = 4 per group. Gating strategy is indicated in Extended Data Fig. 1.**b**, INU1-IgG or INU1-F(ab)_2_, but not the monovalent INU1-fab trigger platelet aggregation in vitro (all antibody derivatives were added at 10 µg ml^−1^ f.c.; shown are representative traces for *n* = 10 each). **c**, Only multivalent CLEC-2 ligands, such as the snake venom rhodocytin or bivalent INU1-IgG, but not INU1-fab trigger CLEC-2 signaling in vitro as assessed by a tyrosine phosphorylation western blot (4G10). Newly phosphorylated proteins are indicated by arrows, the hash symbol indicates the band originating from the detection of INU1-fab by the secondary antibody. The depicted blot is representative of three independent experiments. **d**–**f**, Administration of INU1-F(ab)_2_ (0.5 µg g^−1^) or INU1-IgG (0.75 µg g^−1^) results in immediate platelet consumption, while INU1-fab (0.5 µg g^−1^) results in slowly progressing platelet consumption in vivo; depicted are mean ± s.d. (**d**). In contrast, in vivo only INU1-fab, but not INU1-F(ab)_2_ or INU1-IgG result in neurological impairment, like seizures (**e**) and is associated with lethality; untreated *n* = 5 (for all time points, except for 0 min where *n* = 7), INU1-IgG; *n* = 6–10, INU1-F(ab)_2_; *n* = 5–10, INU1-fab; *n* = 6–10, biologically independent (**f**). *n* = 5–10 mice per group; see source data files for exact numbers. Source data

The anti-CLEC-2 antibody, INU1 (ref. ^9^), or divalent F(ab)_2_ fragments thereof trigger activation of mouse platelets in vitro (Fig. 1a–c), whereas the monovalent INU1-fab has no detectable activatory effect as shown by aggregometry, flow cytometry, scanning electron microscopy (SEM) and protein tyrosine phosphorylation analysis^9^ (Fig. 1a–c). To test the effect of direct CLEC-2/ITAM activation in vivo, mice received INU1-IgG (0.75 µg g^−1^), INU1-F(ab)_2_ or INU1-fab (each 0.5 µg g^−1^) intravenously and were monitored. During the first minutes, INU1-IgG or F(ab)_2_-treated animals consistently were less active with signs of forced breathing, but rapidly recovered and exhibited no further impairment for up to 10 days. Peripheral platelet counts (pPCs) in both groups were reduced by more than 95% at 5 min after injection and remained below 30% of normal for at least 24 h, indicating instantaneous systemic platelet activation and consumption^14^ (Fig. 1d). In sharp contrast, all INU1-fab-treated mice consistently developed a dramatic neurological phenotype within 10 min starting with reduced exploratory activity, impaired motor control of the hind limbs and backward bending of the head and backside. This was followed by focal and generalized tonic–clonic jerks often throwing the animals to one side indicative of generalized seizure activity (Fig. 1e and Supplementary Video 1). Between these attacks, mice moved slowly or laid silently and flaccid on the cage bottom, either not responding to touch or displaying stimulus-induced myoclonic jerks until forced breathing intensified and 77% of the mice died within 45 min (Fig. 1f). The surviving mice were still in very poor condition, displaying repeated seizures and hypothermia and were therefore euthanized after 5 h for animal welfare reasons. At the onset of behavioral abnormalities, pPCs were reduced to 71% and further declined to 39% of baseline after 20 min, demonstrating slowly progressing platelet consumption (Fig. 1d).

**Fig. 2:**
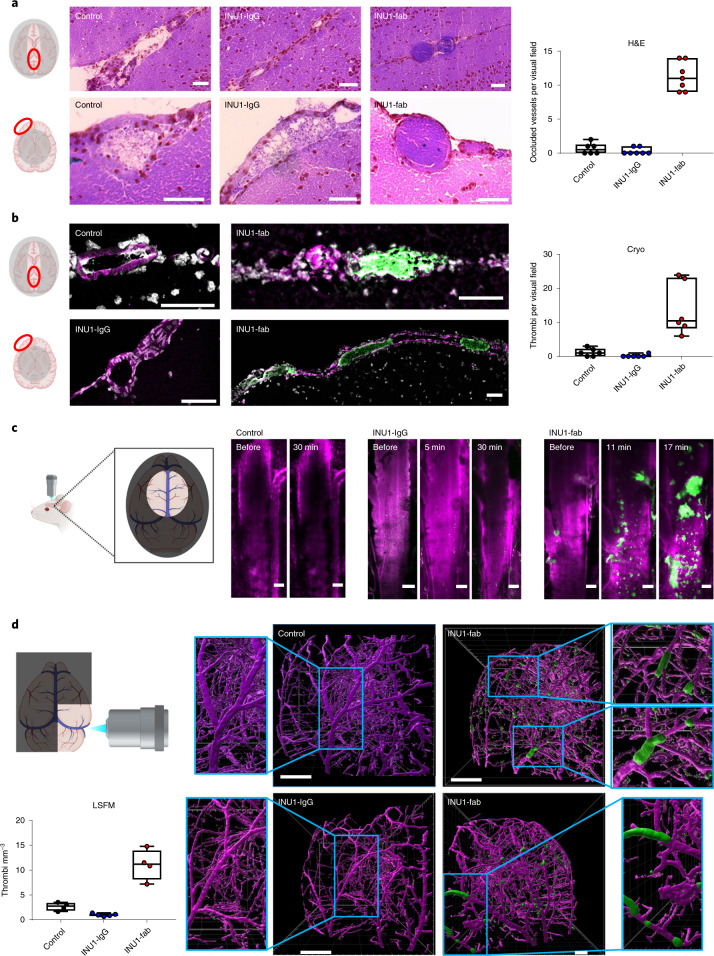
INU1-fab causes cerebral thrombosis. **a**,**b**, Horizontal sections of murine brains (scale 50 µm) taken 20 min after vehicle (control; *n* = 6), INU1-IgG (0.75 µg g^−1^; *n* = 7) or INU1-fab (0.5 µg g^−1^; *n* = 7 and 6, respectively) i.v. injection reveal cerebral thrombi predominantly located in large vessels in between the two hemispheres (upper panels), or at the outside of the cortex (lower panels). Thrombi were visualized using H&E (**a**) or anti-GPIX (p0p6^15^; green counterstained with anti-CD31, magenta and 4,6-diamidino-2-phenylindole, gray) on cryo-sections (Cryo) (**b**). **c**, A cranial window was mounted on top of the superior sagittal sinus and blood flow and thrombus formation were monitored using intravital confocal microscopy. Platelets were stained using an anti-GPIX derivative^15^ (green), the endothelial lining was stained with anti-CD31 and the vessel lumen with fluorescently labeled bovine serum albumin (both depicted in magenta); scale 100 µm. Shown are snapshots from intravital microscopy videos (Supplementary Videos 2–4) at the indicated time points after i.v. injection of vehicle (control; *n* = 3), INU1-IgG (0.75 µg g^−1^; *n* = 4) or INU1-fab (0.5 µg g^−1^; *n* = 6). **d**, Three-dimensional reconstruction of LSFM images of brains from mice that were perfusion-fixed 20 min after i.v. application of vehicle (control; *n* = 4), INU1-IgG (0.75 µg g^−1^; *n* = 5) or INU1-fab (0.5 µg g^−1^; *n* = 4). Platelets (anti-GPIX derivative^15^, green) and endothelial cells (anti-CD31, anti-CD105, both in magenta) were stained in vivo, paraformaldehyde-fixed brains were cleared using benzyl alcohol/benzyl benzoate and imaged on a custom-built light-sheet fluorescence microscope^15^; the scale is indicated as a grid with a size of 500 µm (Supplementary Videos 5–8). Numerical data are displayed as box whisker blots with median being displayed as central line and whiskers indicating minimum and maximum. Source data

The conspicuous neurobehavioral phenotype in conjunction with platelet consumption in INU1-fab-treated mice pointed to the brain as the main injury site and thrombosis as a possible underlying mechanism. To investigate this, mice received either INU1-IgG (0.75 µg g^−1^), INU1-fab (0.5 µg g^−1^), or vehicle and organs were prepared for histopathologic examination after 20 min. Hematoxylin and eosin (H&E)-stained brain sections of INU1-fab-treated mice revealed numerous occluded veins/venules, mostly apparent on the surface (Fig. 2a), reminiscent of CVT in humans. Immunohistochemical staining^15^ confirmed that the vessel occluding thrombi were platelet rich (Fig. 2b). No overt parenchymal tissue damage, for example hemorrhages or edema formation, was seen at this early time point. In contrast, only low numbers of single platelets, but no platelet aggregates, were found in the brains of phosphate-buffered saline (PBS)-treated or INU1-IgG-treated mice (Fig. 2a,b).

**Extended Data Fig. 2:**
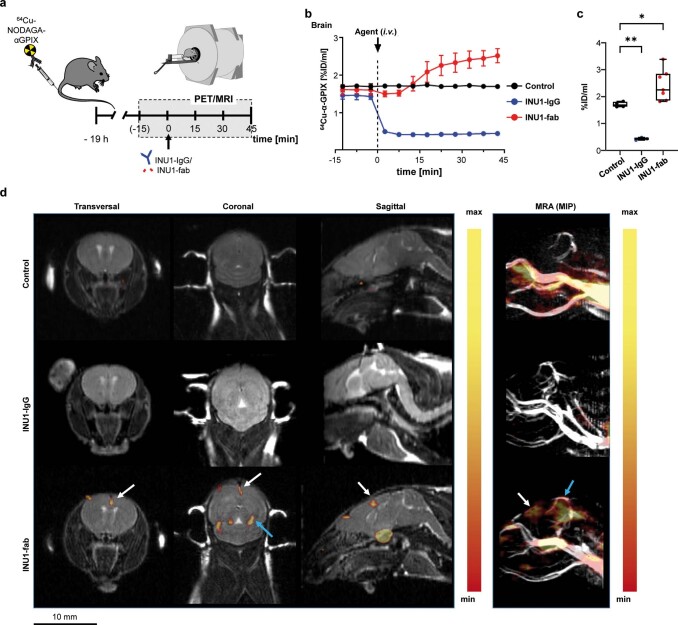
In vivo detection of accumulating platelets in the brain after administration of INU1-fab. **a**. Experimental setup of the simultaneous PET/MRI measurements using [^64^Cu]Cu-NODAGA-anti-GPIX-derivative (64Cu-αGPIX) to detect accumulating platelets. ^64^Cu-αGPIX was injected i.v. into naive C57BL/6J mice. After 19 h of conscious uptake, mice were measured via simultaneous PET/MRI. PET acquisition (60 min) was started directly or 15 min after i.v. injection of INU1-IgG (0.75 µg/g; n = 3), INU1-fab (0.5 µg/g; n = 7) or NaCl (n = 4), which was used as a vehicle control. **b, c**. In vivo quantification represented by time activity curves (TAC) of the ^64^Cu-αGPIX-PET signal in the brain (b) and quantitative analysis of the last 20 min of acquisition (4 × 5 min time frames) of the ^64^Cu-αGPIX-PET signal in the brain (c). In (b) the data is presented as mean ± SEM and in (c) the median is displayed as central line, while the whiskers indicate minimum and maximum. **d**. Representative PET/MR images of the brain from the respective treated mice showing the last 20 min of acquisition (left) and corresponding maximum intensity projections (MIP) of the PET/2D-time of flight magnetic resonance angiograms (MRA, right) showing the vessels in the head. *p = 0.0381 **p = 0.0048; One-way ANOVA followed by Dunnett’s test for multiple comparisons. Colored arrows are highlighting the same thrombi in different planes of the brain. Source data

To map further the rapid dynamics of thrombus formation, we radiolabeled the platelet-specific anti-GPIX monoclonal antibody (mAb) derivative^15^ with copper-64 (^64^Cu-αGPIX) and scanned the mice 19 h after its injection (intravenous (i.v.), 5 µg) for 60 min using simultaneous positron-emission tomography (PET)/magnetic resonance imaging (MRI). INU1-IgG/fab was administered intravenously during PET/MRI acquisition (Extended Data Fig. 2a). Within 45 min, a gradual accumulation of the ^64^Cu-αGPIX signal was noted in the brain exclusively after INU1-fab administration (+0.95 ± 0.50% ID ml^−1^, *n* = 7). Control treatment represents the baseline signal from circulating platelets in the vessels/blood flow of the brain with a negligible difference over time (+0.12 ± 0.09% ID ml^−1^, *n* = 4; Extended Data Fig. 2b,d). Quantitative analysis of the last 20 min of the ^64^Cu-αGPIX signal in the brain revealed strong platelet accumulation after INU1-fab treatment (2.43 ± 0.22% ID ml^−1^, *P* < 0.04; Extended Data Fig. 2c) compared to control treatment (1.17 ± 0.05% ID ml^−1^). Importantly, after co-localization of the ^64^Cu-αGPIX-PET signal with simultaneously acquired two-dimensional (2D)-time of flight (TOF) magnetic resonance angiography (MRA) of the cerebral vessels, we could clearly assign the signal to the (sinus) veins (Extended Data Fig. 2d). After injection of INU1-IgG, loss of circulating platelets in the brain was immediately detectable by the fast and markedly reduced ^64^Cu-αGPIX-PET signal. Next, we utilized intravital microscopy through a cranial window to assess the superior sagittal sinus, a predilection site for CVT in humans^2^ that is lost during routine preparations after removal of the skull (Fig. 2c and Supplementary Videos 2–4). Starting at 7–12 min after INU1-fab injection, rapidly evolving thrombus formation was consistently observed in the superior sagittal sinus (thus shortly preceding the onset of neurological symptoms) which progressed until death of the animal (Supplementary Video 2). In contrast, no thrombotic activity was seen on injection of vehicle or INU1-IgG, but the loss of circulating platelets was noted in the latter group (Supplementary Videos 3 and 4). Changes in blood flow were not observed in these animals for up to 90 min (Supplementary Videos 3 and 4).

**Extended Data Fig. 3:**
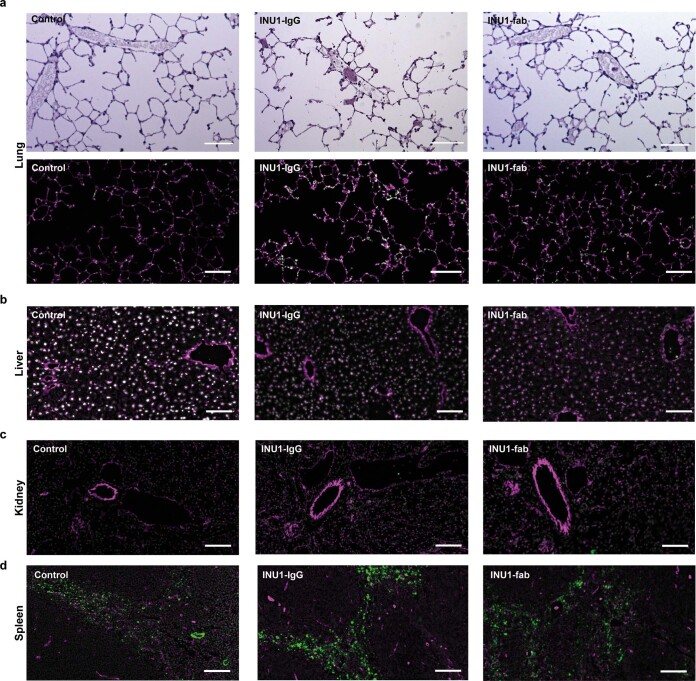
INU1-derivatives do not trigger disseminated thrombosis. Cryo-sections of different murine organs (scale 100 µm) taken 20 min after vehicle (control; n = 6), INU1-IgG (0.75 µg/g; n = 6) or INU1-fab (0.5 µg/mL; n = 6) i.v. injection. Thrombi/platelets were visualized using hematoxylin and eosin (a, upper panels - lung) or anti-GPIX, green – counter-stained with anti-CD31, magenta and DAPI, gray; a-d. Representative sections of lung (a), liver (b), kidney (c) and spleen (d).

Light-sheet fluorescence microscopy (LSFM) of entire hemispheres confirmed the widespread appearance of occlusive thrombi in numerous, even deep large veins of the brains in INU1-fab, but not INU1-IgG or vehicle-treated mice (Fig. 2d and Supplementary Videos 5–8). We also examined additional organs (lung, liver, kidney, spleen) by immunohistochemistry (Extended Data Fig. 3). In contrast to the brain, increased numbers of single platelets but sparsely and generally small aggregates were seen in the lungs of INU1-fab-treated mice at *t* = 20 min (Extended Data Fig. 3a). In all three groups, livers and kidneys were free of platelet aggregates and only a few single platelets were detectable (Extended Data Fig. 3b,c). However, in the lungs of INU1-IgG-treated animals, small platelet aggregates were detected at *t* = 20 min (Extended Data Fig. 3a), which did, however, not cause major pulmonary problems.

### CVT is driven by cooperative CLEC-2 and GPIIb/IIIa signaling

**Fig. 3:**
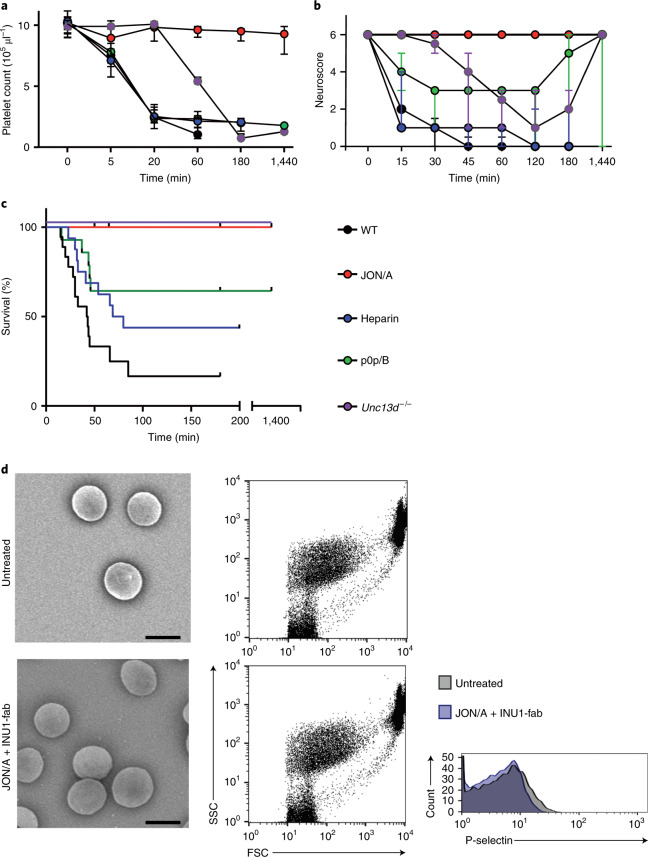
INU1-fab-induced platelet consumption and CVT strictly require functional GPIIb/IIIa. **a**, Platelet counts (median and interquartile range (IQR)) were monitored using flow cytometry at the indicated time points after INU1-fab (0.5 µg g^−1^, i.v.) treatment in the depicted mutant or treated wild-type (WT) mice. Heparin (2 U g^−1^, Ratiopharm, Heparin-Natrium-5000 intraperitoneally (i.p.)), JON/A-F(ab’)_2_ (2 µg g^−1^, i.v.) or p0p/B-fab (2 µg g^−1^, i.v.) were administered 30 min before INU1-fab. WT *n* = 7–13, heparin *n* = 4–16, JON/A *n* = 5–13, p0p/B *n* = 5–14, *Unc13d*^*−/−*^ *n* = 5–6, biologically independent (see source data file for exact numbers for each time point). **b**, Neurological symptoms of the indicated mice were assessed at different time points using a six-point scoring system (Supplementary Video 9) and depicted as median and IQR: 0, death; 1, severe seizures or circling behavior; 2, post-seizure lethargy; 3, mouse lying ‘exhausted’ on the belly; 4, backward bending of the head and backside; 5, reduced motor control of the hind limbs; 6, seemingly unaffected behavior. WT *n* = 13, heparin *n* = 16, JON/A *n* = 14, p0p/B *n* = 14, *Unc13d*^*−/−*^ *n* = 10, biologically independent. **c**, Mortality following INU1-fab challenge was monitored in the indicated groups. WT *n* = 14, heparin *n* = 16, JON/A *n* = 14, p0p/B *n* = 14, *Unc13d*^*−/−*^ *n* = 10, biologically independent. **d**, SEM (panels on the left-hand side, scale 2 µm) and flow cytometry (panels on the right-hand side) demonstrate that platelets in JON/A-treated mice (2 µg g^−1^, i.v.) are resting 20 min post INU1-fab (0.5 µg g^−1^, i.v.) treatment. Depicted are representative images and flow cytometry data of one mouse per group representing *n* = 3 per group for electron microscopy and *n* = 8 for flow cytometry (pooled from two independent experiments); FSC indicates forward scatter and SSC side scatter. Source data

**Extended Data Fig. 4:**
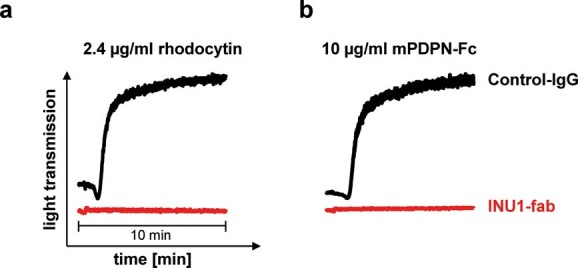
INU1-fab prevents rhodocytin- and podoplanin-induced platelet activation. Pretreatment with 10 µg/mL INU1-fab (for 5 min at 37 °C) prevented rhodocytin-induced (a, 2.4 µg/ml f.c.) or podoplanin (b, 10 µg/ml murine-podoplanin-Fc) platelet aggregation. Depicted are representative aggregometry traces of n = 5 per group.

**Extended Data Fig. 5:**
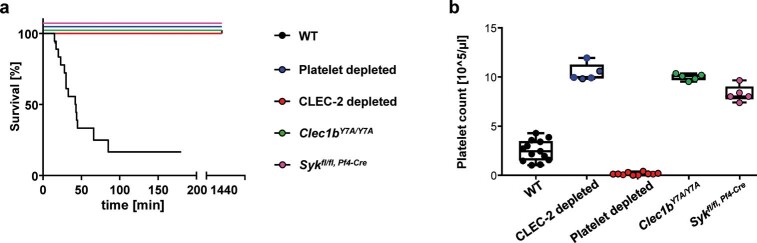
INU1-fab induced platelet consumption and mortality strictly require platelet CLEC-2/ITAM signaling. **a**. Mortality following INU1-fab (0.5 µg/g i.v.) challenge was monitored in the indicated groups. WT n = 14, all other groups n = 10, biologically independent. **b**. Platelet counts were determined 20 min after INU1-fab treatment of the depicted mice using flow cytometry. The results are depicted in a box whisker blot with the central line representing the median and the whiskers indicating minimum and maximum. WT n = 13, mutant and CLEC-2 depleted mice n = 5, platelet-depleted n = 10, biologically independent. Source data

**Extended Data Fig. 6:**
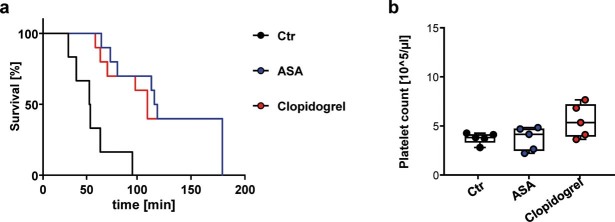
Acetyl salicylic acid (ASA) or Clopidogrel delay INU1-fab induced neurological symptoms, but do not prevent lethality. **a**. Mortality following INU1-fab (0.5 µg/g i.v.) challenge was monitored in the indicated groups. Ctr n = 6, ASA n = 10, Clopidogrel n = 6, biologically independent. **b**. Platelet counts were determined 20 min after INU1-fab treatment of the depicted mice using flow cytometry. n = 5, biologically independent. The results are depicted in a box whisker blot with the central line representing the median and the whiskers indicating minimum and maximum. Source data

Given the rather platelet inhibitory effect of INU1-fab on CLEC-2 in vitro (Extended Data Fig. 4), the mechanistic basis for its pathogenic effect in vivo was unclear and was thus studied further^9^. Notably, previous platelet depletion (R300-antibody) completely protected mice from INU1-fab-induced neurological symptoms and lethality (Extended Data Fig. 5). Similarly, mice depleted of CLEC-2 (ref. ^9^) or expressing CLEC-2 with a mutated, signaling-dead hemITAM in blood cells (*Clec1b*^*Y7A/Y7A*^)^16^ or lacking spleen tyrosine kinase (*Syk*^*fl/fl, Pf4-Cre*^)^17^, an essential component of the CLEC-2-ITAM signaling pathway, were resistant to INU1-fab-induced platelet consumption, neurological impairment and lethality (Extended Data Fig. 5) demonstrating a key role of CLEC-2/ITAM signaling for the pathogenesis of CVT in these animals. ‘Classic’ platelet-dependent thrombus formation involves the sequential action of different adhesion receptors and the autocrine/paracrine effects of released secondary mediators^18^. GPIb mediates platelet recruitment into growing thrombi and has been implicated in arterial and venous thrombosis^19–21^. Interestingly, functional inhibition of GPIb by p0p/B-fab^22^ did not prevent INU1-fab-induced platelet consumption (Fig. 3a), but partly mitigated the development of neurological symptoms (Fig. 3b). Accordingly, still five of 14 mice died but the remaining mice recovered from neurological symptoms within 24 h (Fig. 3b,c). Platelet activation leads to the release of dense and α-granule content, which amplifies the activation response and promotes thrombus formation. We therefore assessed INU1-fab-induced CVT in *Unc13d*^*−/−*^ mice, which display abolished platelet-dense granule release and markedly impaired α-granule release^23^. Interestingly, in these mice platelet consumption after INU1-fab treatment was comparable to wild-type (WT*)* controls, although with a significantly delayed onset (Fig. 3a). Despite the consistent appearance of neurological symptoms, and the overall bad shape of these mice within the first 4 h, their condition thereafter stabilized and all (10/10) survived and completely recovered from neurological symptoms within 24 h. In contrast, all INU1-fab-treated WT controls died (Fig. 3b,c). This motivated us to investigate the efficacy of classic platelet inhibitors clopidogrel (P2Y_12_ ADP-receptor blocker) or acetylsalicylic acid (ASA) in preventing INU1-fab-induced cerebral venous (sinus) thrombosis (CV(S)T). Notably, both treatments delayed the onset of symptoms, but did not prevent thrombocytopenia or lethality (Extended Data Fig. 6). Activated platelets promote thrombin generation and thrombin, in turn, is a powerful platelet agonist and the central protease of the coagulation cascade that produces fibrin^24^. Thrombin is a key factor in the pathogenesis of VT and is targeted by different anticoagulants^4^. Heparin is a widely used indirect thrombin inhibitor and is currently the recommended first-line treatment for patients with CVT, even in the setting of venous congestion-induced intracranial hemorrhages^1,2,25^. Therefore, we pretreated mice with heparin (2 U g^−1^.) followed by INU1-fab (0.5 µg g^−1^) after 20 min. Heparin had no effect on INU1-fab-induced platelet consumption (Fig. 3a) and most of the animals developed neurological symptoms, although with slightly delayed onset. Acute mortality was reduced (7/16 survived the first 3 h; Fig. 3b,c). After 5 h, these mice were still alive, but consistently displayed severe lethargy, seizures, hypothermia and ruffled fur, and were euthanized for animal welfare reasons.

**Extended Data Fig. 7:**
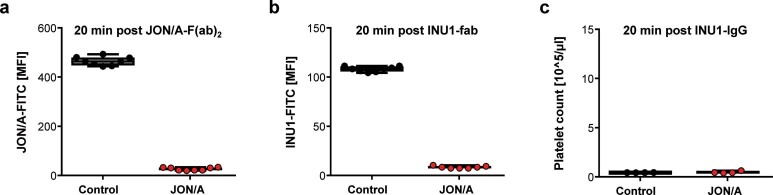
GPIIb/IIIa-bockade does not prevent INU1-IgG induced platelet consumption. **a**. GPIIb/IIIa-blockade by JON/A-F(ab)_2_ treatment (2.0 µg/g i.v.) was confirmed by the absence of JON/A^FITC^ binding using flow cytometry 20 min after JON/A-F(ab)_2_-treatment (n = 8 mice per group). **b**. Flow cytometric analysis of platelets isolated from untreated WT mice (control, n = 8) and mice pre-treated with JON/A-F(ab)_2_ (2.0 µg/g; 10 min before INU1-fab challenge, n = 7) 20 min after INU1-fab treatment (0.5 µg/g, i.v.). Absence of INU1^FITC^ binding confirms the presence of INU1-fab on the circulating platelets despite unaltered platelet counts in these animals (see Fig. 3a). **c**. In contrast to INU1-fab induced platelet consumption, GPIIb/IIIa-blockade (2.0 µg/g JON/A-F(ab)_2_ i.v.) does not prevent INU1-IgG induced platelet consumption, as demonstrated by the markedly reduced platelet counts of vehicle or JON/A-F(ab)_2_-pretreated animals 20 min after INU1-IgG (0.75 µg/g i.v.; n = 4 mice per group). Each symbol represents one individual, the box plots depict median and minimum to maximum. Source data

Platelet activation results in the shift of GPIIb/IIIa from a low to a high affinity state (inside-out signaling), a step referred to as the ‘final common pathway’ of platelet activation^26^. High affinity GPIIb/IIIa binds fibrinogen and other ligands leading to platelet aggregation. Ligand-occupied GPIIb/IIIa, in turn, transmits signals into the platelet (outside-in signaling) and thereby controls different cellular responses, including spreading and clot retraction^27^. GPIIb/IIIa blockers are the most powerful inhibitors of platelet function and have been in clinical use for decades to prevent in-stent thrombosis following percutaneous coronary intervention^4,28^. We pretreated mice with F(ab)_2_ of the GPIIb/IIIa blocking antibody, JON/A^22,29^ (2 µg g^−1^), followed by injection of INU1-fab (0.5 µg g^−1^) after 30 min. Remarkably, all (12/12) JON/A-pretreated mice appeared indistinguishable from healthy PBS-injected controls for the entire observation period (3 h) and showed no neurological impairment (Fig. 3b,c). Further, pPCs remained unchanged in these mice (Fig. 3a) although INU1-fab was detectable on the platelet surface (Extended Data Fig. 7). Flow cytometry and SEM confirmed that these circulating platelets were in a resting state (Fig. 3c), suggesting that functional GPIIb/IIIa not only mediates INU1-fab-induced aggregate formation in vivo but is also essentially required for the initiation of platelet activation in this pathological setting. Based on these results, we speculate that GPIIb/IIIa blockers could be more protective in acute CVT than ASA or P2Y_12_ ADP-receptor blockers. Notably, JON/A-pretreated mice were monitored for one week and no signs of bleeding or delayed CVT were observed. JON/A pretreatment did not prevent the rapid drop in pPCs in INU1-IgG-treated mice (Extended Data Fig. 7), indicating that these fully (CLEC-2/ITAM)-activated platelets were cleared GPIIb/IIIa independently, presumably by splenic phagocytes.

### Therapeutic GPIIb/IIIa blockade protects from CVT and death

**Fig. 4:**
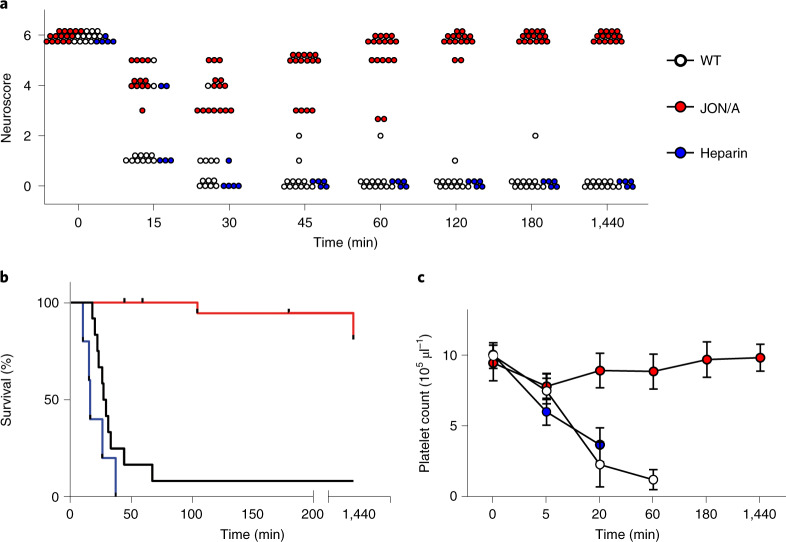
GPIIb/IIIa blockade, but not heparin administration, after symptom onset prevents INU1-fab-induced platelet consumption CVT and death. Mice were treated with INU1-fab (0.5 µg g^−1^ intravenously) and after disease onset received vehicle, heparin (2 U g^−1^) or JON/A-F(ab)_2_ (2 µg g^−1^) intravenously. **a**, Neurological symptoms of the indicated mice were assessed at different time points using a six-point scoring system (Supplementary Video 9): 0, death; 1, severe seizures or circling behavior; 2, post-seizure lethargy; 3, mouse lying ‘exhausted’ on the belly; 4, backward bending of the head and backside; 5, reduced muscle control of the hind limbs; 6, seemingly unaffected behavior (Supplementary Video 10).Vehicle control *n* = 12, heparin *n* = 5, JON/A *n* = 15, biologically independent. **b**, Mortality following INU1-fab challenge was monitored in the indicated groups. Vehicle control *n* = 12, heparin *n* = 6, JON/A *n* = 15–20, biologically independent. **c**, Platelet counts (mean ± s.d.) were monitored using flow cytometry at the indicated time points in the depicted mice. For gating strategy see Extended Data Fig. 1. Vehicle *n* = 7–12, heparin *n* = 5, JON/A *n* = 5–15, biologically independent (see source data file for exact numbers for each time point). Source data

To compare the effects of heparin and GPIIb/IIIa inhibition in a therapeutic setting, WT mice received INU1-fab (0.5 µg g^−1^) and were monitored for first neurological symptoms, which consistently occurred after 8 ± 2 min (reduced activity, tonic deflection of the head and backside; Fig. 4a). Two minutes after symptom onset, mice received either heparin (2 U g^−1^), JON/A-F(ab)_2_ (2 µg g^−1^), or vehicle intravenously. As expected, in all vehicle-treated mice the neurological symptoms and seizures were aggravated and 11/12 mice died within 70 min (Fig. 4b). Similarly, the heparin-treated mice also displayed aggravating neurological symptoms and all (5/5) died within 38 min (Fig. 4a,b). Of note, platelet consumption had progressed after heparin treatment (pPC: 37% of baseline at 20 min after INU1-Fab; Fig. 4c). In clear contrast, while all JON/A-F(ab)_2_-treated mice showed reduced activity (score 3), no other signs of neurological impairment or seizures were seen at any time point after intervention and they fully recovered within 2–3 h (Fig. 4a and Supplementary Video 10). Of note, pPCs in these mice remained stable or even slightly increased compared to the time point of intervention (Fig. 4c), suggesting that the treatment instantaneously interrupted platelet activation and consumption. These animals were monitored for one week and showed normal behavior, excluding a delayed disease manifestation under treatment.

We have shown that the induction of aberrant platelet CLEC-2/ITAM signaling is sufficient to induce a rapidly progressing thrombotic syndrome mimicking human CV(S)T. The foudroyant clinical picture is particularly reminiscent of CVT in young patients with coronavirus disease 2019 (COVID-19) with a mortality of up to 40%^30,31^. While anticoagulation is the mainstay of antithrombotic treatments of VT and CVT^2,32^, platelets increasingly get into focus as critical players also in VT^5^. Most recently, platelet activating antibodies against platelet factor 4 have been reported in patients who had developed thrombotic thrombocytopenia and CV(S)T following COVID vaccination^33–35^. Such antibodies trigger platelet activation through clustering of the ITAM-coupled Fc-gamma receptor (FcγR) IIA (CD32) which signals through a similar pathway as CLEC-2^36,37^.

Emerging evidence suggests immune-related platelet activating mediators in the plasma of patients with COVID-19, triggering FcγRIIA-ITAM and other signaling pathways^38^. Mouse platelets lack FcγRIIA^37^ making it difficult to investigate a possible link of this pathway and the development of CVT in vivo. Our data do, however, clearly point to pathological platelet activation as a possible trigger of CVT as ligation of CLEC-2 (via INU1-fab) very selectively directed thrombotic activity to the cerebral venous system. We therefore hypothesize that INU1-fab alters the conformation of CLEC-2 and thereby facilitates its interaction with a (yet to be identified) ligand that appears to be enriched in cerebral veins. GPIIb/IIIa may be required in this setting to facilitate/stabilize the interaction of CLEC-2 with this putative ligand. Although the exact mechanism underlying this selectivity remains to be determined, a systemically accessible agonist triggering direct ITAM-receptor clustering may not drive CVT, as shown by the absence of cerebral thrombotic activity in INU1-IgG-treated animals (Fig. 2). Similarly, anti-GPVI-IgGs trigger ITAM-dependent platelet activation but do not induce thrombosis in mice^36,39,40^. Instead, only the cooperative signaling of (ligated) CLEC-2 and GPIIb/IIIa triggered a platelet activation mechanism that resulted in foudroyant CVT. Thus, CLEC-2 could be a so-far overseen target receptor contributing to immune-related pathological platelet activation ultimately leading to CVT and should be further investigated in patients with COVID-19/CVT. Besides autoimmune antibodies, also soluble CLEC-2 binding partners could trigger the pathogenic effect, and podoplanin is a candidate molecule, as it is strongly upregulated in different tissues during inflammation, including the lung, and can be shed from the cell surface to circulate in plasma^41,42^. Of note, a correlation of angiotensin-converting enzyme 2 (ACE2), the severe acute respiratory syndrome coronavirus 2 entry receptor, and podoplanin was recently reported for human lung tissue^43^ and podoplanin was linked to VT^44^.

Remarkably, blocking GPIIb/IIIa prevented disease development in mice and increased survival on application even after disease onset, while heparin, the first-line treatment for CVT in humans^2,25^, was only partially effective. Thus, GPIIb/IIIa antagonists, or inhibitors of CLEC-2-ITAM signaling, might be considered as an ultimate therapeutic option in foudroyant CVT not controlled by heparin.

## Methods

### Mice and treatments

Mice were maintained under specific pathogen-free conditions (constant temperature of 20–24 °C and 45–65% humidity with a 12-h light–dark cycle, free access to water and food) and experiments were performed in accordance with German law and the governmental bodies, and with approval from the District of Lower Franconia and Tübingen. C57Bl/6J mice were purchased from Charles River; *Clec1b*^*Y7A/Y7A*^, *Syk*^*fl/fl, Pf4-Cre*^ and *Unc13d*^*−/−*^ were described previously^16,17,23^, and kept in our animal house.

INU1-antibody derivatives^9,14^ (0.5 µg g^−1^ body weight of INU1-fab or F(ab)_2_ or 0.75 µg g^−1^ INU1-IgG), platelet-depletion antibodies (Emfret Analytics, R300, 2 µg g^−1^)^45^, JON/A-F(ab)_2_ fragments^22,29^ (2 µg g^−1^) and p0p/B-fab^20^ (2 µg g^−1^) were administered intravenously under isoflurane anesthesia. Unfractionated heparin (Ratiopharm, heparin-5000, 2 U g^−1^) was administered intraperitoneally for the pretreatment setting and intravenously in the therapeutic setting.

Mice were recorded using a standard webcam and Supplementary Videos 1, 9 and 10 were generated using DaVinci Resolve v.17.2 software.

### Platelet flow cytometry and aggregometry

Washed blood was diluted 1:20 and incubated with appropriate fluorophore-conjugated monoclonal antibodies (indicated below) for 15 min at room temperature and analyzed on a FACSCalibur instrument (Becton Dickinson) using the CellQuest Pro (v.6.0) software. For platelet counts anti-GPIbα^FITC^ (p0p4) and anti-GPIIb/IIIa^PE^ (JON6) and for platelet activation anti-P-selectin^FITC^ (WUG1.9) and JON/A^PE^ (Emfret Analytics) were used. Binding of INU1 derivatives, as well as blockade of GPIbα (p0p/B) or GPIIb/IIIa (JON/A) were confirmed using INU1^FITC^, p0p/B^FITC^ or JON/A^FITC^, respectively. Data were analyzed using FlowJo (v.10.7).

Washed platelets (200 µl with 0.25 × 10^6^ platelets µl^−1^) were analyzed in the presence of 70 µg ml^−1^ human fibrinogen (catalog no. F3879, Sigma-Aldrich). Light transmission was recorded on a Fibrintimer 4-channel aggregometer (APACT Laborgeräte und Analysensysteme) for 10 min and was expressed in arbitrary units with buffer representing 100% transmission.

### SEM

Mice were bled from the retro-orbital plexus into a tube containing 20 U ml^−1^ heparin (Ratiopharm). Platelet-rich plasma was obtained by centrifugation at 300*g* for 6 min at room temperature (RT). For the preparation of washed platelets, platelet-rich plasma was centrifuged at 1,000*g* for 5 min and the pellet was suspended and washed once in modified Tyrodes-N-2-hydroxyethlpiperazine-N-2-ethanesulfonicacid (HEPES, 5 mM glucose, 0.35% bovine serum albumin (BSA), pH 7.4) buffer supplemented with prostaglandin I_2_ (0.1 μg ml^−1^, catalog no. 538925 (VWR), Sigma-Aldrich) and apyrase (0.02 U ml^−1^, catalog no. A7646, Sigma-Aldrich). Before analysis, platelets were suspended in the same buffer (0.02 U ml^−1^ apyrase) and allowed to rest for at least 30 min at 37 °C.

Washed platelets (250,000 µl^−1^ final concentration [f.c.]) were incubated with 10 µg ml^−1^ INU1-fab, INU1-F(ab)_2_ or INU1-IgG, respectively, in vitro for 15 min or left untreated. Samples were fixed in solution with 2× Karnovsky buffer for 5 min at 37 °C before fixation on poly-l-lysin coated coverslides (catalog no. P4707, Sigma-Aldrich) for 1 h at RT followed by over night fixation at 4 °C with 1× Karnovsky buffer (2.5% glutaraldehyde in 100 mM cacodylate buffer). On the next day, samples were fixed with 4% paraformaldehyde (PFA)/0.4% glutaraldehyde for 20 min at 4 °C and washed twice with distilled water for 5 min, 4 °C. After washing with 100 mM cacodylate buffer for 5 min, 4 °C, samples were dehydrated using an increasing ethanol alcohol (EtOH) series (4 × 5 min EtOH 75%, 5 min EtOH 80%, 5 min EtOH 95%, all at 4 °C and 2 × 20 min EtOH 100% at RT). Next, samples were incubated with increasing hexamethyldisilazane (HMDS) (catalog no. 3840.2, Carl Roth) concentrations (5 min 25% HMDS diluted in 100% EtOH, 5 min 50% HMDS, 5 min 75% HMDS and twice 5 min 100% HMDS, all at RT). Afterwards, samples were dried by evaporating HMDS, mounted, sputter coated with Au/Pd and examined under a scanning electron microscope (Phenom Pro, Thermofisher Scientific).

### Tyrosine phosphorylation

Washed platelets (500,000 µl^−1^ f.c.) were prepared as described above and incubated with 2.4 µg ml^−1^ rhodocytin, 10 µg ml^−1^ INU1-fab, INU1-F(ab)_2_ or INU1-IgG, respectively, in vitro for 15 min or left untreated. Subsequently, platelets were solubilized in lysis buffer (TRIS-buffered saline containing 15 mM TRIS, 155 mM NaCl, 1 mM EDTA, 0.005% NaN_3_, protease inhibitor cocktail (catalog no. P8340, Sigma-Aldrich) and 0.5% IGEPAL CA-630 (catalog no. I3021, Sigma-Aldrich)). Samples were separated by sodium dodecyl sulfate polyacrylamide gel electrophoresis with a molecular weight marker and transferred onto a polyvinylidene difluoride membrane (Millipore) and probed with anti-pan-phosphotyrosine antibody (Merck/Millipore, catalog no. 05-321, clone 4G10, 1:1,000).

### Histology and immune fluorescence

#### Collection of mouse organs for histology

Mice were euthanized by an overdose of isoflurane, the diaphragm was opened and the lungs were exposed by opening the rib cage. The trachea was cannulated with a 22 G venous catheter (BD Insyte) and 0.8 ml of a mixture containing optimum cutting temperature compound (Tissue-Tek, Sakura, TTEK, Hartenstein) and 10% sucrose in 1× PBS at a ratio of 1:1 was introduced into the collapsed lungs. The trachea was tied up, lungs were harvested en bloc, rinsed in 1× PBS and the heart was removed. Furthermore, the left lobe of the liver, kidney, spleen and brain were harvested en bloc as well and rinsed in 1× PBS. The organs were carefully blotted dry on a paper towel, placed into Tissue-Tek Cryomolds (Sakura), embedded in optimum cutting temperature compound and snap frozen in isopentane (catalog no. 277258, Sigma-Aldrich) precooled on dry ice. Blocks were stored at −80 °C, cross-sections were cut at 7 µm on a Leica CM1950 cryostat (Leica) and mounted on Superfrost Plus slides (Thermo Scientific).

#### H&E and immunofluorescence staining of cryosections

For H&E staining, cryosections of liver and lung were thawed at RT for 10 min and immediately fixed with 4% PFA (pH 7.2, Sigma-Aldrich) for 10 min. Afterwards, sections were washed three times with 1× PBS, followed by a short wash in deionized water. Slides were stained for 30 s in hematoxylin (hematoxylin solution, Gill no. 3, catalog no. GHS332, Sigma-Aldrich) and blueing was performed under a running tap water for 10 min. Thereafter, sections were stained with acidified 0.05% Eosin G solution (catalog no. X883.1, Carl Roth) for 3 min and shortly rinsed in deionized water. Dehydration was carried out by immersion of the sections in an increasing ethanol series (2 min 70% EtOH, 2 min 80% EtOH, 2 min 90% EtOH, 2 min 96% EtOH and 2 min 100% EtOH) and two changes of xylene for 5 min each. Slides were dried under the fume hood, sections were afterwards mounted with Eukitt (catalog no. 03989, Sigma-Aldrich) and kept at RT overnight before imaging.

For immunofluorescence staining of liver, lung, kidney, spleen and brain cryosections, slides were thawed, fixed and washed as mentioned above. Unspecific binding was blocked by incubating the sections with 5% BSA (Fraction V, Carl Roth) and 10 µg ml^−1^ Fc-block (2.4G2) in 1× PBS for 30 min at RT. Fluorescently labeled antibodies were sequentially incubated (5 µg ml^−1^ in 1% BSA and 0.1% Tween-20 in 1× PBS) at RT for 60 min each. Tissue sections were washed three times with the antibody dilution buffer after each antibody incubation step. Alexa Fluor 546-labeled anti-GPIX derivative^15^ and anti-CD31 Alexa Fluor 647 (clone 390, BioLegend) were used to stain platelets and endothelium, respectively. After three final washing steps, sections were mounted with Fluoroshield with 4,6-diamidino-2-phenylindole (catalog no. F6057, Sigma-Aldrich) to stain nuclei. Slides were kept at 4 °C overnight before imaging.

#### Imaging of histological sections

Images of H&E or immunofluorescent stained sections were acquired on a Leica Thunder Imager DMi8, equipped with a Leica DFC9000GT fluorescence and an Andor Zyla 4.2 bright field camera, using a ×10 or ×20 objective and the Leica Application Suite (LAS) X software (v.3.7). Deconvolution was performed on the fly using the LAS X Thunder software. For quantification, the number of cerebral thrombi was counted in three separate horizontal sections per animal and were used for statistical analysis (symbols in Fig. 2 represent biological replicates, not different sections). Representative micrographs of the different organs were processed and visualized using Fiji^46^.

### PET imaging

#### Preparation of radiolabeled anti-GPIX

Anti-GPIX mAb derivative (anti-GPIX) was conjugated with the metal chelator 2,2′-(7-(1-carboxy-4-((4-isothiocyanatobenzyl)amino)-4-oxobutyl)-1,4,7-triazonane-1,4-diyl)diacetic acid (p-NCS-benzyl-NODAGA) and radiolabeled with in-house produced radioactive ^64^Cu (Department of Preclinical Imaging and Radiopharmacy, University of Tubingen, Tubingen, Germany) using a labeling ratio of 2:1 (2 MBq [^64^Cu]CuCl_2_ per µg of NODAGA-anti-GPIX). The labeling efficacy of the radiolabeled [^64^Cu]Cu-NODAGA-anti-GPIX (referred to as ^64^Cu-αGPIX) was determined by high performance liquid chromatography and thin layer chromatography.

#### Simultaneous PET/MRI

Female 9-week-old C57BL/6J mice were used for simultaneous PET/MRI measurements. Mice were anesthetized using 1.5% of isoflurane (5% for induction vaporized in 100% oxygen) followed by i.v. injection of 5 µg ^64^Cu-αGPIX into the tail vein. Afterwards the mice were put back into their cages for conscious radiotracer uptake 19 h after the i.v. injection of ^64^Cu-αGPIX, the mice were anesthetized as described above. A catheter was placed into the tail vein of the mice to enable the administration of INU1-IgG/fab (or NaCl as control) during PET/MRI acquisition. Next, the animals were placed onto a warmed small-animal bed and positioned into the center field of view (FoV) of the MRI scanner (7 T ClinScan, Bruker Bio Spin MRI). Short localizer magnetic resonance protocols were applied to adjust the position of the mice in the FoV of the scanner. To acquire ^64^Cu-αGPIX-PET simultaneously with MRI, a small-animal PET insert was installed permanently inside the magnetic resonance scanner as described previously^47^.

Simultaneous PET/MRI measurements were performed over the course of 60 min. The respiration rate was monitored during the whole acquisition. INU1-IgG/fab or NaCl were administered once the PET scan was started or 15 min afterwards. Standard three-dimensional anatomical magnetic resonance protocols of the brain and the body were used for anatomical references. For a precise visualization of the vessels, a non-contrast-enhanced 2D-TOF MRA was acquired using the following image parameters: slice thickness 0.41 mm, in-plane spatial resolution 0.156 × 0.156 mm^2^, imaging matrix 128 × 128, FoV 20 × 20 mm^2^, flip angle 80°, echo time 2.695 ms, repetition time sequence 18 ms. A maximum intensity projection was reconstructed based on the TOF angiography. PET data were stored as list-mode files and reconstructed in 12 × 5 min frames using an ordered-subsets expectation maximization 2 algorithm including random, decay, time-delay corrections and normalization. The algorithm is based on the reconstruction software of the Inveon PET Scanner (Siemens Healthcare) and has been adapted to the geometrical configuration of the PET insert.

#### PET/MRI image analysis

Analysis of the ^64^Cu-αGPIX signal in the brain was performed using Inveon Research Workplace software (Siemens Preclinical Solutions). Fusion of the reconstructed PET images, magnetic resonance images and 2D-TOF MRAs was performed and volumes of interest of the brain were created based on the anatomical magnetic resonance images. ^64^Cu-αGPIX accumulation in the volumes of interest was given in Bq ml^−1^. The reported values represent the mean activity concentration expressed as the percentage injected dose per ml of tissue (% ID ml^−1)^ and were decay-corrected to the imaging time point. For a visual comparison of the PET images, the signal intensities have been adjusted to each other based on the injected dose indicated by common color look-up tables.

#### Statistical analysis of PET imaging datasets

Data are presented as mean ± s.e.m. and box plots as median (95% confidence intervals). For statistical analysis, one-way analysis of variance (ANOVA) followed by Dunnett’s test for multiple comparisons was performed using GraphPad Prism software (v.9.0.2). Differences of *P* <0.05 were considered significant (*P < 0.05; **P < 0.01).

### Intravital imaging

Before imaging, an open cranial window was induced into the skull. To this end, mice were anesthetized by intraperitoneal injection of medetomidine (0.5 μg g^−1^, Pfizer), midazolam (5 μg g^−1^, Roche) and fentanyl (0.05 μg g^−1^, Janssen-Cilag). At the site of surgery, hair was removed, and the skin was disinfected with 70% isopropyl alcohol. Skin was removed to expose the skull. To remove the bone on top of the superior sagittal sinus, a circle with a diameter of approximately 5 mm was cut between the bregma and lambda using a high-speed microdrill. To avoid overheating, the skull was repeatedly cooled with saline. The cut skull region was removed using forceps without damaging the dura. Subsequently, the exposed superior sagittal sinus was covered with a small drop of saline and a sterile, round coverslip (diameter 6 mm) that was fixed with cyanoacrylate glue. The exposed skull was covered with dental cement. To allow i.v. injection during microscopy, a catheter was placed in the jugular vein. The vasculature was visualized by injection of BSA Alexa Fluor 546 (8 µg g^−1^ body weight) and anti-CD105 Alexa Fluor 546 (clone MJ7/19, purified in-house, 0.4 µg g^−1^ body weight). Platelets were visualized by injection of Alexa Fluor 488-labeled anti-GPIX derivative^15^ (0.6 µg g^−1^). Then, a custom designed metal plate was fixed to the open cranial window and the mouse was mounted on a custom-built stereotactic frame to reduce head movement during imaging. Mice were placed under an upright Leica SP8 confocal microscope and imaging was performed using a ×10 objective. After 5 min of imaging, mice were treated with INU1-fab (0.5 µg g^−1^), INU1-IgG (0.75 µg g^−1^) or vehicle without stopping image acquisition. Image stacks were processed, visualized and analyzed using Fiji^46^.

### LSFM

#### Sample preparation

Platelets and the vasculature were stained by injecting Alexa Fluor 750-labeled anti-GPIX derivative^15^ (0.6 µg g^−1^), anti-CD31 Alexa Fluor 647 (BioLegend, clone 390, 0.4 µg g^−1^) and anti-CD105 Alexa Fluor 647 (clone MJ7/19, purified in-house, 0.4 µg ^−1^), respectively, 10 min before administering INU1 derivatives. Twenty minutes after injection of INU1 derivatives, mice were transcardially perfused with ice-cold PBS to wash out the blood and subsequently with ice-cold 4% PFA (pH 7.2, P6148, Sigma-Aldrich) to fix the tissues. Brains were harvested and stored in 4% PFA for 30 min. Samples were then washed in PBS, followed by dehydration in a graded methanol (Sigma-Aldrich) series (50%, 70%, 95%, 100% for 30 min each) at RT and stored at 4 °C overnight. The methanol was replaced stepwise by a clearing solution consisting of one part benzyl alcohol to two parts benzyl benzoate (BABB, catalog nos. 305197 and B6630, Sigma-Aldrich). After incubation in the clearing solution for at least 2 h at RT, tissue specimens became optically transparent and were used for LSFM imaging on the following day.

#### LSFM setup and data acquisition

The LSFM setup is tailor made for organ imaging and custom built. Major parts have been described before^48^. Laser lines were provided by a custom-made laser box. In brief, visible lasers (Obis 488 nm LS 60 mW, Obis 532 nm LS 50 mW, Obis 561 nm LS 50 mW, Obis 640 nm Lx 100 mW, Coherent) were combined with a suitable dichroic beam splitter (593/LP BrightLine, 542/LP BrightLine, 500/LP BrightLine, AHF) to pass an Acousto-Optic Tunable Filter (AA.AOTF.nC-T 642, AA Opto Electronic) and to be coupled into a single mode fiber (kineFLEX-P-3-S-730-0.7-FCP-P2, Qioptiq). The infrared laser (LuxX CW 730 nm 40 mW, Omicron Laserage) was directed to single mode fiber (kineFLEX-P-3-S-730-0.7-FCP-P2, Qioptiq). For excitation both infrared and visible beam paths were collimated with ×10 objectives (UPlanFL N 10x/0.30 for VIS, PlanN 10x/0.25 for 730 nm, Olympus), respectively, and merged via a dichroic beam splitter (DCLP 660, AHF Analysentechnik) and adjusted to fit the beam size and divergence with a beam expander (BE03M-A, Thorlabs) in a de-magnifying arrangement. The beam was elongated by a dual-axis galvanometer scanner (6210H, Cambridge Technologies) to create a virtual light sheet in combination with a theta lens (VISIR, Leica), which is projected onto the ample via a tube lens (model 452960, Zeiss) and the illumination objective (EC Epiplan-Neofluar 2.5/0.06 M27, Zeiss). Alternating two-sided illumination was realized for larger objects. Fluorescence from the sample was collected perpendicularly to the light sheet by a ×5 objective (HC PL Fluotar 5x/0.15, Leica) and an infinity-corrected tube lens (model Valentine RTC, Leica) and projected onto a scientific complementary metal oxide semiconductor camera (Neo 5.5, Andor). The fluorescence was spectrally filtered by suitable emission filters (BrightLine HC 525/50 (Autofluorescence), HQ697/58 (Alexa Fluor 647), BrightLine HC 785/62 (Alexa Fluor 750), AHF Analysentechnik) driven by a motorized filter wheel (HA110A with ES10 controller, Prior Scientific GmbH) placed between the detection objective and tube lens. Samples were mounted on a holder moved by a motorized (8MS00-25-28, Standa) three-axis stage (M-423-MIC, Newport) and placed in a custom-made chamber filled with clearing solution consisting of one part benzyl alcohol (catalog no. 0336.2, Carl Roth GmbH) and of two parts benzyl benzoate (catalog no. B6630-1L, Sigma-Aldrich) (BABB) to match the refractive index. For stack acquisition, the sample was traveled through the light sheet. Hardware components for image acquisition (laser, camera, stage, filter wheel) were controlled by commercial software (IQ v.2.9, Andor).

Image acquisition of a cleared brain samples was performed as a tile scan sequentially with 220 ms exposure time to get a stack of typically 2,048 × 2,048 × 1,200 voxels with voxel size of 1.3 × 1.3 × 5 µm^3^. Images were saved as .tiff files and analyzed as described below.

#### Deconvolution

Platelet (Alexa Fluor 750) and endothelium (Alexa Fluor 647) channels were deconvolved using the batch option in Huygens Professional 20.04 (SVI). For the endothel channel automatic background subtraction was performed using the lowest value within a one-pixel radius, the signal-to-noise ratio was determined as 10 and a maximum of 40 iterations were performed. For the platelet channel, automatic background subtraction was done using the ‘in/near object’ option with a radius of 2 µm (2 pixels). The signal-to-noise ratio was set to 40 and a maximum of 60 iterations were performed. The PSF of our home-built LSFM setup was characterized as follows for the deconvolution: detection numerical aperture NA = 0.15, refractive index 1.554, illumination lens NA = 0.03, fill factor for the illumination lens 0.5, illumination from left and a Gaussian beam profile with a width of 18 µm.

The deconvolved data were converted into the Imaris file format and binned 2× in the *xy* direction to achieve 1,024 × 1,024 pixels per slice.

#### Recognizing autofluorescence by machine learning

The data from the autofluorescence channel collected after 488 nm excitation was binned 2× in the *xy* direction to end up with 1,024 × 1,024 pixels per slice and imported into Ilastik^49^. Ilastik was trained to recognize two classes ‘background’ and ‘autofluorescence’. The respective probabilities for both classes were rescaled from 0–1 to 0–10,000 and exported as 16-bit unit .tiff images. These images were then converted into the Imaris file format.

#### Segmentation

The preprocessed files from the three fluorescence channels were combined into a single Imaris 9.6 file (Bitplane, Oxford Instruments) and further analyzed using its surface segmentation tool. The autofluorescence was segmented using the ‘autofluorescence’ probabilities with an intensity threshold of 5,000 (thus reflecting 50% probability). Smaller, unconnected areas were removed manually. Next, the endothelium channel was segmented using a sample-dependent intensity threshold between 200 and 400 and a size filter of 1,000 voxels. For both channel segmentations, the surface grain size was left at 5.2 µm. The total volume from the autofluorescence reflecting the imaged brain volume and the total volume of the endothelial system were exported. Finally, the thrombi were segmented using a surface grain size of 10 µm, a sample-dependent intensity threshold between 100 and 350 and a volume size filter of 1,000 µm³. Thrombi that were not in contact with the segmented vasculature were excluded from analysis. For each of the remaining and detected thrombi the volume was exported and a size distribution, mean and standard deviation calculated.

### Statistical analyses

No statistical methods were used to predetermine the sample size. Pilot experiments and previously published results were used to estimate the sample size, such that appropriate statistical tests could yield significant results. For the treatment groups mice were randomly assigned cage-wise and experiments were performed in a blinded manner during experiments and outcome assessment. Data were collected in Microsoft Excel (Microsoft Office 365) and statistical analysis was performed using GraphPad Prism software v.7.03 and v.9.0.2 (GraphPad Software).

### Sample sizes, biological replicates and statistical tests

The following values were used.

Figure 1a: *n* = 4 mice per group, scale bar 2 µm. Figure 1b: *n* = 10 mice per group. Figure 1c: *n* = 4–6 mice per group in three independent experiments. Figure 1d: untreated *n* = 5–7, INU1-IgG *n* = 6–10, INU1-F(ab)_2_ *n* = 5–10, INU1-fab *n* = 6–10. Figure 1e: IgG-control *n* = 6, INU1-IgG *n* = 6, INU1-F(ab)_2_ *n* = 6, INU1-fab *n* = 10; Mantel–Cox test: *P* < 0.001. Figure 1f: IgG-control *n* = 6, INU1-IgG *n* = 6, INU1-F(ab)_2_ *n* = 6, INU1-fab *n* = 7; Mantel–Cox test: *P* < 0.001.

Figure 2a: Control *n* = 6, INU1-IgG *n* = 7, INU1-fab *n* = 7; ANOVA with Kruskal–Wallis test: *P* < 0.01 (INU1-fab versus control) and *P* <0.01 (INU1-fab versus INU1-IgG). Figure 2b: *n* = 6 mice per group; ANOVA with Kruskal–Wallis test: *P* < 0.01 (INU1-fab versus control) and *P* < 0.01 (INU1-fab versus INU1-IgG). Figure 2c: Control *n* = 3, INU1-IgG *n* = 4, INU1-fab *n* = 6; Fig. 2d: Control *n* = 4, INU1-IgG *n* = 5, INU1-fab *n* = 4; ANOVA with Kruskal–Wallis test: *P* < 0.01 (INU1-fab versus INU1-IgG).

Figure 3a: WT *n* = 7–13, heparin *n* = 4–16, JON/A *n* = 5–13, p0p/B *n* = 5–14, *Unc13d*^*−/−*^ *n* = 5–6. Figure 3b: WT *n* = 13, heparin *n* = 16, JON/A *n* = 14, p0p/B *n* = 14, *Unc13d*^*−/−*^ *n* = 10. Figure 3c: WT *n* = 14, heparin *n* = 16, JON/A *n* = 14, p0p/B *n* = 14, *Unc13d*^*−/−*^ *n* = 10. Figure 3d: *n* = 3 mice per group for electron microscopy and *n* = 8 for flow cytometry.

Figure 4a: Control *n* = 12, heparin *n* = 5, JON/A *n* = 15. Figure 4b: Control *n* = 12, heparin *n* = 6, JON/A *n* = 15–20. Figure 4c: Control *n* = 7–12, heparin *n* = 5, JON/A *n* = 4–15; Mantel–Cox test: *P* < 0.001.

For exact numbers per time point see source data file.

### Reporting Summary

Further information on research design is available in the Nature Research Reporting Summary linked to this article.

### Supplementary information

Supplementary Information File contains all extended figures with figure legends to provide an overview and figure legends for the supplementary videos

Reporting Summary

Supplementary Video 1 INU1-fab-treated animal. Progression of neurological symptoms after INU1-fab (0.5 µg g^−1^ i.v.) treatment.

Supplementary Video 2 IVM of the superior sagittal sinus post INU1-fab treatment. A cranial window was mounted on top of the superior sagittal sinus and blood flow and thrombus formation were monitored using intravital confocal microscopy. Platelets were stained using an anti-GPIX derivative (green), the endothelial lining was stained with anti-CD105 and the vessel lumen with fluorescently labeled BSA (both depicted in magenta); scale 100 µm. Shown is a representative confocal video from intravital microscopy of a INU1-fab-treated (0.5 µg g^−1^) mouse.

Supplementary Video 3 IVM of the superior sagittal sinus post vehicle treatment. A cranial window was mounted on top of the superior sagittal sinus and blood flow and thrombus formation were monitored using intravital confocal microscopy. Platelets were stained using an anti-GPIX derivative (green), the endothelial lining was stained with anti-CD105 and the vessel lumen with fluorescently labeled BSA (both depicted in magenta); scale 100 µm. Shown is a representative confocal video from intravital microscopy of a vehicle-treated mouse.

Supplementary Video 4 IVM of the superior sagittal sinus post INU1-IgG treatment. A cranial window was mounted on top of the superior sagittal sinus and blood flow and thrombus formation were monitored using intravital confocal microscopy. Platelets were stained using an anti-GPIX derivative (green), the endothelial lining was stained with anti-CD105 and the vessel lumen with fluorescently labeled BSA (both depicted in magenta); scale 100 µm. Shown is a representative confocal video from intravital microscopy of a INU1-IgG-treated (0.75 µg g^−1^) mouse.

Supplementary Video 5 LSFM of a brain hemisphere of an INU1-fab-treated mouse. Three-dimensional (3D) reconstruction of LSFM images of a brain hemisphere from an INU1-fab-treated (0.5 µg g^−1^) mouse. Platelets (anti-GPIX derivative, green) and endothelial cells (anti-CD31, anti-CD105, both in magenta) were stained in vivo, PFA-fixed brains were cleared using benzyl alcohol/benzyl benzoate (BABB) and imaged on a custom-built LSFM.

Supplementary Video 6 LSFM of a brain hemisphere of an INU1-fab-treated mouse. 3D reconstruction of LSFM images of a brain hemisphere from an INU1-fab-treated (0.5 µg g^−1^) mouse. Platelets (anti-GPIX derivative, green) and endothelial cells (anti-CD31, anti-CD105, both in magenta) were stained in vivo, PFA-fixed brains were cleared using BABB and imaged on a custom-built LSFM.

Supplementary Video 7 LSFM of a brain hemisphere of a vehicle-treated mouse. 3D reconstruction of LSFM images of a brain hemisphere from a vehicle-treated mouse. Platelets (anti-GPIX derivative, green) and endothelial cells (anti-CD31, anti-CD105, both in magenta) were stained in vivo, PFA-fixed brains were cleared using BABB and imaged on a custom-built LSFM.

Supplementary Video 8 LSFM of a brain hemisphere of an INU1-IgG-treated mouse. 3D reconstruction of LSFM images of a brain hemisphere from an INU1-IgG-treated (0.75 µg g^−1^) mouse. Platelets (anti-GPIX derivative, green) and endothelial cells (anti-CD31, anti-CD105, both in magenta) were stained in vivo, PFA-fixed brains were cleared using BABB and imaged on a custom-built LSFM.

Supplementary Video 9 Exemplified illustration of the symptoms representing the different levels of the neuroscore. Neurological symptoms of INU1-fab-treated (0.5 µg g^−1^) mice were assessed at different time points using a six-point scoring system that is exemplified in this video: 0, death; 1, severe seizures or circling behavior; 2, post-seizure lethargy; 3, mouse lying ‘exhausted’ on the belly; 4, backward bending of the head and backside; 5, reduced motor control of the hind limbs; 6, seemingly unaffected behavior.

Supplementary Video 10 Comparison of INU1-fab challenged mice having received either heparin pretreatment or therapeutic JON/A-F(ab)_2_ treatment. Shown are sequences of INU1-fab-treated (0.5 µg g^−1^) mice that were either pre-treated with heparin (2 U g^−1^ i.p. 30 min before INU1-fab) or therapeutically treated with JON/A-F(ab)_2_ (2 µg g^−1^ i.v. after symptom onset) at different time points after INU1-fab treatment.

### Source data

Source Data Fig. 1 File contains source data

Source Data Fig. 1 uncropped gels of figure 1c

Source Data Fig. 2 File contains source data

Source Data Fig. 3 File contains source data

Source Data Fig. 4 File contains source data

Source Data Extended Data Fig. 2 File contains source data

Source Data Extended Data Fig. 5 File contains source data

Source Data Extended Data Fig. 6 File contains source data

Source Data Extended Data Fig. 7 File contains source data

## Figures and Tables

**Extended Data Fig. 1:**
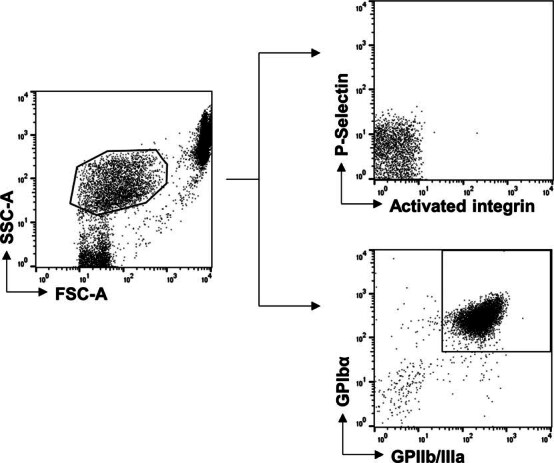
Gating strategy for flow cytometry. Platelets were gated from whole blood according to their FSC, SSC characteristics. Platelet activation was assessed by surface expression of P-Selectin or activated integrin (upper panel). Platelet count was determined by gating on GPIbα, GPIIb/IIIa double positive platelets (lower panel). For washed platelets, gating strategy was the same.

## Data Availability

The datasets generated and/or analyzed during the current study are available in the source data file.
