# Supplementary material for: Foudroyant cerebral venous (sinus) thrombosis triggered through CLEC-2 and GPIIb/IIIa dependent platelet activation
Source: Nat Cardiovasc Res. 2022 Feb 10;1(2):132–41. doi: 10.1038/s44161-021-00017-1 (PMC11358028; doi:10.1038/s44161-021-00017-1)
Supplement: Supplementary file 1 — File contains all extended figures with figure legends to provide an overview and figure legends for the supplementary videos [file 44161_2021_17_MOESM1_ESM.pdf]

nature portfolio

https://doi.org/10.1038/s44161-021-00017-1

Supplementary information

Foudroyant cerebral venous (sinus) thrombosis triggered through CLEC-2 and GPIIb/IIIa dependent platelet activation

In the format provided by the
authors and unedited

Supplementary Material

Foudroyant cerebral venous (sinus) thrombosis triggered through CLEC-2 and GPIIb/IIIa dependent platelet activation

David Stegner[1,2,*], Vanessa Göb[1,*], Viola Krenzlin[1,2,3], Sarah Beck[1,2], Katherina Hemmen[2], Michael K. Schuhmann[4], Barbara F. Schörg[5], Christian Hackenbroch[1], Frauke May[1,2,6], Philipp Burkard[1,2], Jürgen Pinnecker[2], Alma Zernecke[1], Peter Rosenberger[7], Andreas Greinacher[8], Bernd J. Pichler[5,9], Katrin G. Heinze[2], Guido Stoll[4], and Bernhard Nieswandt[1,2,§]

[1]Institute of Experimental Biomedicine, University Hospital, University of Würzburg, Würzburg, Germany. [2]Rudolf Virchow Center, University of Würzburg, Würzburg, Germany. [3]Current address: Center for Thrombosis and Hemostasis, Johannes Gutenberg University Medical Center, Mainz, Germany; [4]Department of Neurology, University Hospital Würzburg, [5]Werner Siemens Imaging Center, Department of Preclinical Imaging and Radiopharmacy, Eberhard Karls University of Tübingen, Tübingen, Germany; [6]Current address: CSL Behring Innovation GmbH, Marburg, Germany; [7]Department of Anesthesiology and Intensive Care Medicine, University Hospital, Tübingen, Germany, [8]Institute of Immunology and Transfusion Medicine, University Medicine Greifswald, Germany; [9]Cluster of Excellence iFIT (EXC 2180) "Image-Guided and Functionally Instructed Tumor Therapies", Eberhard Karls University Tübingen, Tübingen, Germany.

*both authors contributed equally

Correspondence to: Bernhard Nieswandt, PhD; Institute of Experimental Biomedicine, University Hospital and Rudolf Virchow Center, University of Würzburg; Josef-Schneider-Str. 2, 97080 Würzburg, Germany; Phone: + 49 931 31 80405; Fax: + 49 931 201 61652; E-mail: bernhard.nieswandt@virchow.uni-wuerzburg.de

Extended Figures

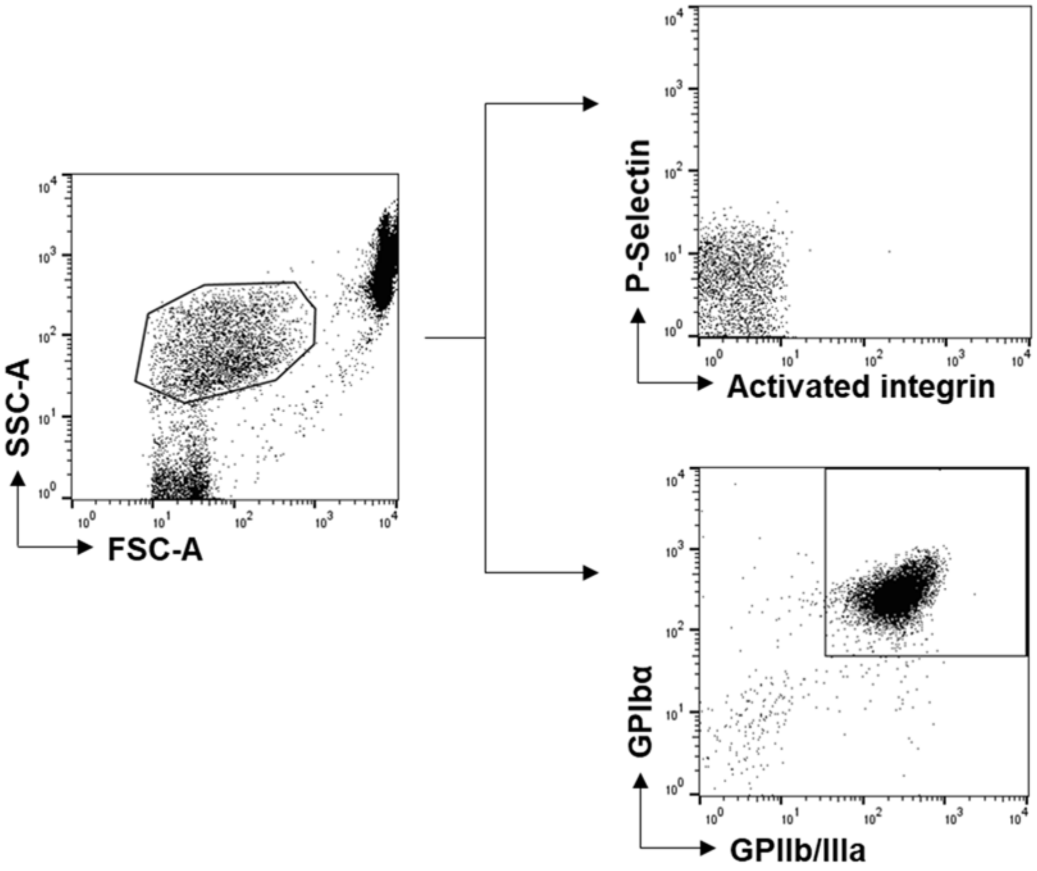

Extended Fig. 1 │ Gating strategy for flow cytometry. Platelets were gated from whole blood according to their FSC, SSC characteristics. Platelet activation was assessed by surface expression of P-Selectin or activated integrin (upper panel). Platelet count was determined by gating on GPIbα, GPIIb/IIIa double positive platelets (lower panel). For washed platelets, gating strategy was the same.

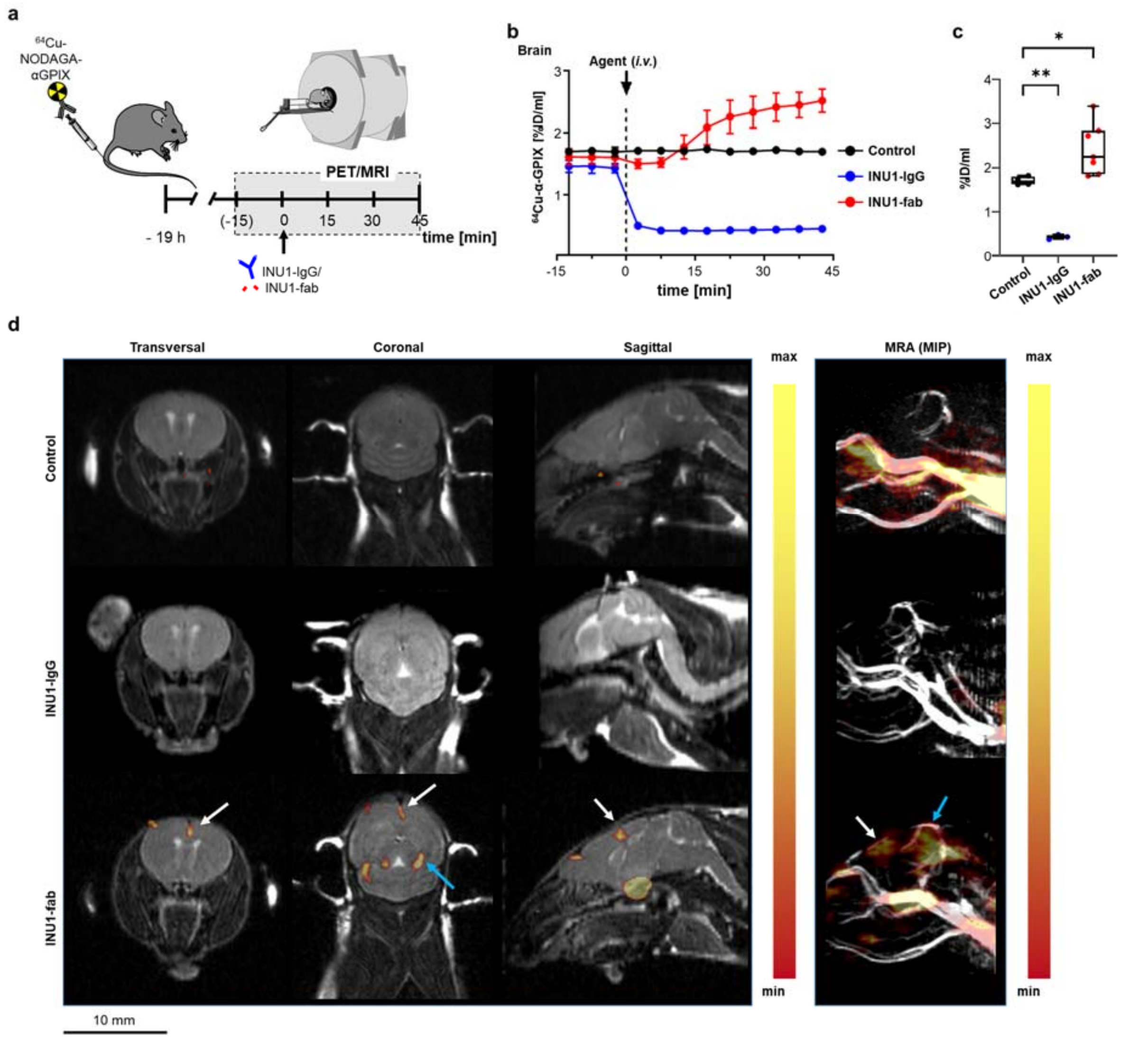

Extended Fig. 2 │ In vivo detection of accumulating platelets in the brain after administration of INU1-fab. **a.** Experimental setup of the simultaneous PET/MRI measurements using [^{64}Cu]Cu-NODAGA-anti-GPIX-derivative (^{64}Cu-αGPIX) to detect accumulating platelets. ^{64}Cu-αGPIX was injected i.v. into naive C57BL/6J mice. After 19 h of conscious uptake, mice were measured via simultaneous PET/MRI. PET acquisition (60 min) was started directly or 15 min after i.v. injection of INU1-IgG (0.75 µg/g; n=3), INU1-fab (0.5 µg/g; n=7) or NaCl (n=4), which was used as a vehicle control. **b, c.** In vivo quantification represented by time activity curves (TAC) of the ^{64}Cu-αGPIX-PET signal in the brain (**b**) and quantitative analysis of the last 20 min of acquisition (4x5 min time frames) of the ^{64}Cu-αGPIX-PET signal in the brain (**c**). In (**b**) the data is presented as mean ± SEM and in (**c**) the median is displayed as central line, while the whiskers indicate minimum and maximum. **d.** Representative PET/MR images of the brain from the respective treated mice showing the last 20 min of acquisition (left) and corresponding maximum intensity projections (MIP) of the PET/2D-time of flight magnetic resonance angiograms (MRA, right) showing the vessels in the

head. *p = 0.0381 **p = 0.0048; One-way ANOVA followed by Dunnett's test for multiple comparisons. Colored arrows are highlighting the same thrombi in different planes of the brain.

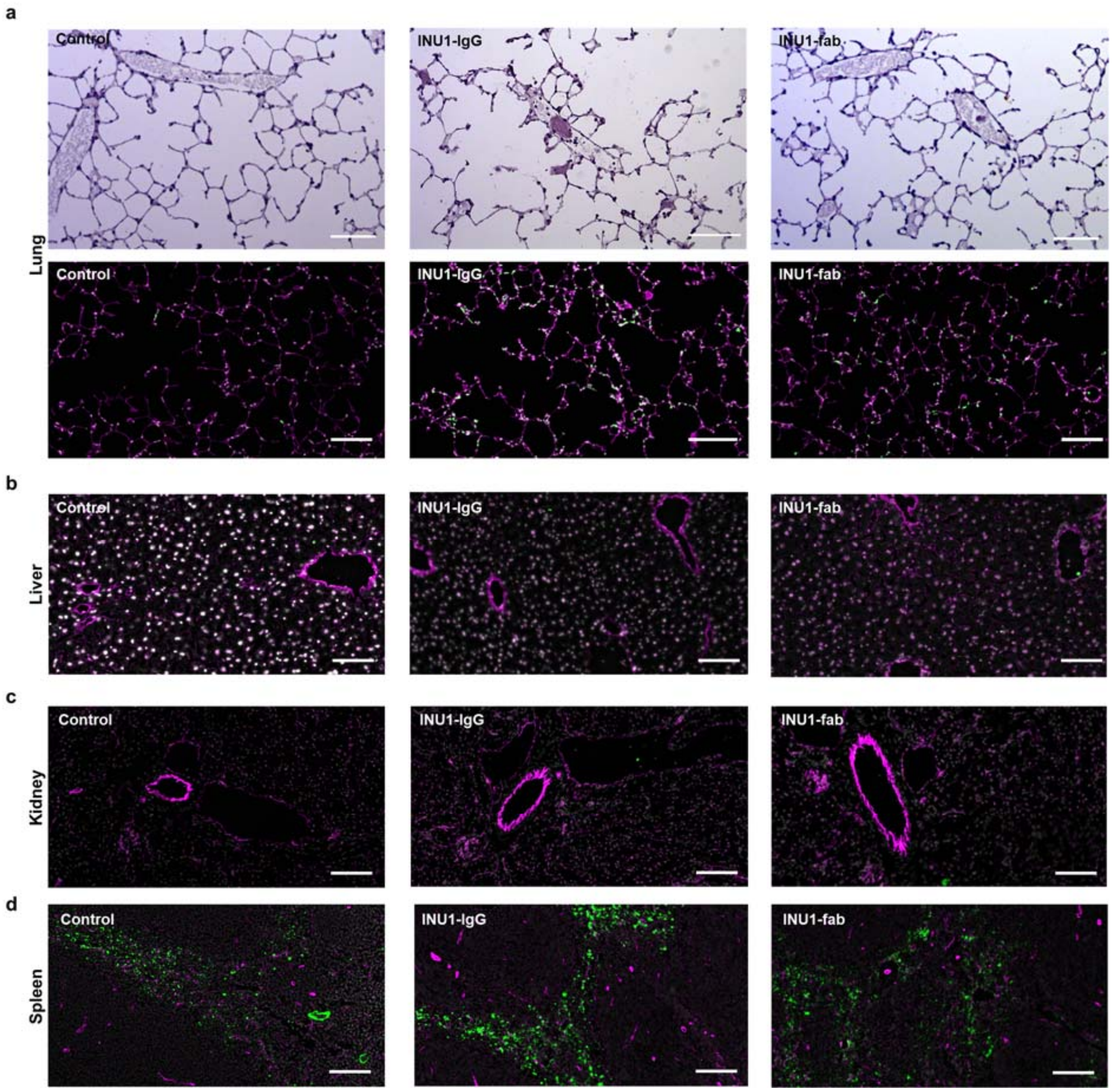

Extended Fig. 3 │ INU1-derivatives do not trigger disseminated thrombosis. Cryo-sections of different murine organs (scale 100 µm) taken 20 min after vehicle (control; n=6), INU1-IgG (0.75 µg/g; n=6) or INU1-fab (0.5 µg/mL; n=6) i.v. injection. Thrombi/platelets were visualized using hematoxylin and eosin (**a**, upper panels - lung) or anti-GPIX, green – counter-stained with anti-CD31, magenta and DAPI, gray; **a-d**. Representative sections of lung (**a**), liver (**b**), kidney (**c**) and spleen (**d**).

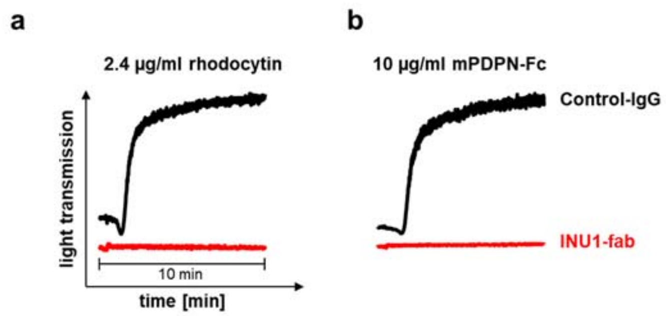

Extended Fig. 4 │ INU1-fab prevents rhodocytin- and podoplanin-induced platelet activation. Pretreatment with 10 µg/mL INU1-fab (for 5 min at 37°C) prevented rhodocytin-induced (**a**, 2.4 µg/ml f.c.) or podoplanin (**b**, 10 µg/ml murine-podoplanin-Fc) platelet aggregation. Depicted are representative aggregometry traces of n=5 per group.

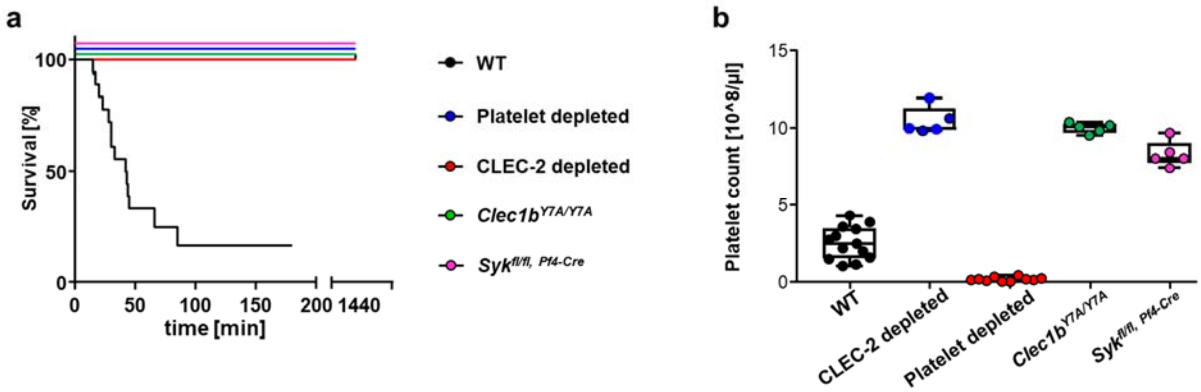

Extended Fig. 5 │ INU1-fab induced platelet consumption and mortality strictly require platelet CLEC-2/ITAM signaling. a. Mortality following INU1-fab (0.5 µg/g i.v.) challenge was monitored in the indicated groups. WT n=14, all other groups n=10, biologically independent. **b.** Platelet counts were determined 20 min after INU1-fab treatment of the depicted mice using flow cytometry. The results are depicted in a box whisker blot with the central line representing the median and the whiskers indicating minimum and maximum. WT n=13, mutant and CLEC-2 depleted mice n=5, platelet-depleted n=10, biologically independent.

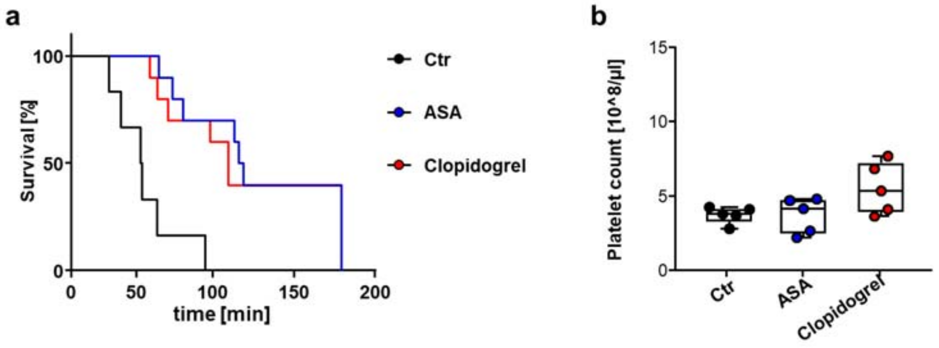

Extended Fig. 6 │ Acetyl salicylic acid (ASA) or Clopidogrel delay INU1-fab induced neurological symptoms, but do not prevent lethality. a. Mortality following INU1-fab (0.5 µg/g i.v.) challenge was monitored in the indicated groups. Ctr n=6, ASA n=10, Clopidogrel n=6, biologically independent. **b.** Platelet counts were determined 20 min after INU1-fab treatment of the depicted mice using flow cytometry. n=5, biologically independent. The results are depicted in a box whisker blot with the central line representing the median and the whiskers indicating minimum and maximum.

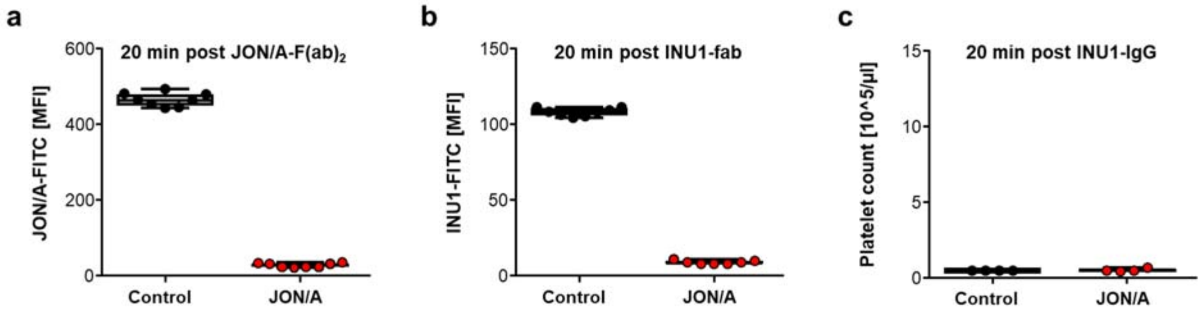

Extended Fig. 7 │ GPIIb/IIIa-bockade does not prevent INU1-IgG induced platelet consumption. a. GPIIb/IIIa-blockade by JON/A-F(ab)$_2$ treatment (2.0 µg/g i.v.) was confirmed by the absence of JON/A^{FITC} binding using flow cytometry 20 min after JON/A-F(ab)$_2$-treatment (n=8 mice per group). **b**. Flow cytometric analysis of platelets isolated from untreated *WT* mice (control, n=8) and mice pre-treated with JON/A-F(ab)$_2$ (2.0 µg/g; 10 min before INU1-fab challenge, n=7) 20 min after INU1-fab treatment (0.5 µg/g, i.v.). Absence of INU1^{FITC} binding confirms the presence of INU1-fab on the circulating platelets despite unaltered platelet counts in these animals (see Fig. 3a). **c.** In contrast to INU1-fab induced platelet consumption, GPIIb/IIIa-blockade (2.0 µg/g JON/A-F(ab)$_2$ i.v.) does not prevent INU1-IgG induced platelet consumption, as demonstrated by the markedly reduced platelet counts of vehicle or JON/A-F(ab)$_2$-pretreated animals 20 min after INU1-IgG (0.75 µg/g i.v.; n=4 mice per group). Each symbol represents one individual, the box plots depict median and minimum to maximum.

Supplementary Videos

Video 1 - INU1-fab treated animal. Progression of neurological symptoms after INU1-fab (0.5 µg/g i.v.) treatment.

Video 2 - IVM of the superior sagittal sinus post INU1-fab treatment. A cranial window was mounted on top of the superior sagittal sinus and blood flow and thrombus formation were monitored using intravital confocal microscopy. Platelets were stained using an anti-GPIX derivative (green), the endothelial lining was stained with anti-CD105 and the vessel lumen with fluorescently labeled BSA (both depicted in magenta); scale 100 µm. Shown is a representative confocal video from intravital microscopy of a INU1-fab-treated (0.5 µg/g) mouse.

Video 3 - IVM of the superior sagittal sinus post vehicle treatment. A cranial window was mounted on top of the superior sagittal sinus and blood flow and thrombus formation were monitored using intravital confocal microscopy. Platelets were stained using an anti-GPIX derivative (green), the endothelial lining was stained with anti-CD105 and the vessel lumen with fluorescently labeled BSA (both depicted in magenta); scale 100 µm. Shown is a representative confocal video from intravital microscopy of a vehicle-treated mouse.

Video 4 - IVM of the superior sagittal sinus post INU1-IgG treatment. A cranial window was mounted on top of the superior sagittal sinus and blood flow and thrombus formation were monitored using intravital confocal microscopy. Platelets were stained using an anti-GPIX derivative (green), the endothelial lining was stained with anti-CD105 and the vessel lumen with fluorescently labeled BSA (both depicted in magenta); scale 100 µm. Shown is a representative confocal video from intravital microscopy of a INU1-IgG-treated (0.75 µg/g) mouse.

Video 5 - LSFM of a brain hemisphere of an INU1-fab treated mouse. 3D reconstruction of light-sheet fluorescence microscopy (LSFM) images of a brain hemisphere from an INU1-fab-treated (0.5 µg/g) mouse. Platelets (anti-GPIX derivative, green) and endothelial cells (anti-CD31, anti-CD105, both in magenta) were stained in vivo, PFA-fixed brains were cleared using benzyl alcohol/benzyl benzoate (BABB) and imaged on a custom-built LSFM.

Video 6 - LSFM of a brain hemisphere of an INU1-fab treated mouse. 3D reconstruction of light-sheet fluorescence microscopy (LSFM) images of a brain hemisphere from an INU1-fab-treated (0.5 µg/g) mouse. Platelets (anti-GPIX derivative, green) and endothelial cells (anti-CD31, anti-CD105, both in magenta) were stained in vivo, PFA-fixed brains were cleared using benzyl alcohol/benzyl benzoate (BABB) and imaged on a custom-built LSFM.

Video 7 - LSFM of a brain hemisphere of a vehicle treated mouse. 3D reconstruction of light-sheet fluorescence microscopy (LSFM) images of a brain hemisphere from a vehicle-treated mouse. Platelets (anti-GPIX derivative, green) and endothelial cells (anti-CD31, anti-CD105, both in magenta) were stained in vivo, PFA-fixed brains were cleared using benzyl alcohol/benzyl benzoate (BABB) and imaged on a custom-built LSFM.

Video 8 - LSFM of a brain hemisphere of an INU1-IgG treated mouse. 3D reconstruction of light-sheet fluorescence microscopy (LSFM) images of a brain hemisphere from an INU1-IgG-treated (0.75 µg/g) mouse. Platelets (anti-GPIX derivative, green) and endothelial cells (anti-CD31, anti-CD105, both in magenta) were stained in vivo, PFA-fixed brains were cleared using benzyl alcohol/benzyl benzoate (BABB) and imaged on a custom-built LSFM.

Video 9 - Exemplified illustration of the symptoms representing the different levels of the neuroscore. Neurological symptoms of INU1-fab-treated (0.5 µg/g) mice were assessed at different time points using a six-point scoring system that is exemplified in this video: 0 = death, 1 = severe seizures or circling behavior, 2 = post-seizure lethargy, 3 = mouse lying 'exhausted' on the belly, 4 = backward bending of the head and backside, 5 = reduced motor control of the hind limbs, 6 = seemingly unaffected behavior.

Video 10 - Comparison of INU1-fab challenged mice having received either heparin pre-treatment or therapeutic JON/A-F(ab)$_2$ treatment. Shown are sequences of INU1-fab-treated (0.5 µg/g) mice that were either pre-treated with heparin (2 U/g i.p. 30 min before INU1-fab) or therapeutically treated with JON/A-F(ab)$_2$ (2 µg/g i.v. after symptom onset) at different time points after INU1-fab treatment.
