# Supplementary material for: Foudroyant cerebral venous (sinus) thrombosis triggered through CLEC-2 and GPIIb/IIIa dependent platelet activation
Source: Nat Cardiovasc Res. 2022 Feb 10;1(2):132–41. doi: 10.1038/s44161-021-00017-1 (PMC11358028; doi:10.1038/s44161-021-00017-1)
Supplement: Supplementary file 2 — Reporting Summary [file 44161_2021_17_MOESM2_ESM.pdf]

nature portfolio

Corresponding author(s): Bernhard Nieswandt

Last updated by author(s): 12/16/2021

# Reporting Summary

Nature Portfolio wishes to improve the reproducibility of the work that we publish. This form provides structure for consistency and transparency in reporting. For further information on Nature Portfolio policies, see our Editorial Policies and the Editorial Policy Checklist.

## Statistics

For all statistical analyses, confirm that the following items are present in the figure legend, table legend, main text, or Methods section.

| n/a | Confirmed | |
|---|---|---|
| ☐ | ☒ | The exact sample size ($n$) for each experimental group/condition, given as a discrete number and unit of measurement |
| ☐ | ☒ | A statement on whether measurements were taken from distinct samples or whether the same sample was measured repeatedly |
| ☐ | ☒ | The statistical test(s) used AND whether they are one- or two-sided<br>*Only common tests should be described solely by name; describe more complex techniques in the Methods section.* |
| ☐ | ☒ | A description of all covariates tested |
| ☐ | ☒ | A description of any assumptions or corrections, such as tests of normality and adjustment for multiple comparisons |
| ☐ | ☒ | A full description of the statistical parameters including central tendency (e.g. means) or other basic estimates (e.g. regression coefficient) AND variation (e.g. standard deviation) or associated estimates of uncertainty (e.g. confidence intervals) |
| ☐ | ☒ | For null hypothesis testing, the test statistic (e.g. $F$, $t$, $r$) with confidence intervals, effect sizes, degrees of freedom and $P$ value noted<br>*Give P values as exact values whenever suitable.* |
| ☒ | ☐ | For Bayesian analysis, information on the choice of priors and Markov chain Monte Carlo settings |
| ☒ | ☐ | For hierarchical and complex designs, identification of the appropriate level for tests and full reporting of outcomes |
| ☒ | ☐ | Estimates of effect sizes (e.g. Cohen's $d$, Pearson's $r$), indicating how they were calculated |

*Our web collection on statistics for biologists contains articles on many of the points above.*

## Software and code

Policy information about availability of computer code

**Data collection**

Data were collected in Microsoft Excel (Microsoft Office 365 for Enterprise).

**Data analysis**

Flow cytometry
Platelets were analyzed using BD FACSCalibur and the CellQuestPro (v6.0) software, gating was based on FSC/SSC characteristics and data were analyzed using FlowJo (v10.7).

Imaging of histological sections.
Images of H&E or immunofluorescent stained sections were acquired on a Leica Thunder Imager DMi8 using the Leica Application Suite (LAS) X software (version 3.7). Deconvolution was performed on the fly using the LAS X Thunder software. Representative micrographs of the different organs were processed and visualized using Fiji (Schindelin et al., Nat Meth 2012).

PET/MRI image analysis.
Analysis of the 64Cu-αGPIX signal in the brain was performed using Inveon Research Workplace software (Siemens Preclinical Solutions, Knoxville, TN, USA). Fusion of the reconstructed PET images, MR images and 2D-TOF MR angiograms was performed and volumes of interest (VOIs) of the brain were created based on the anatomical MR images.

Intravital imaging
Immunofluorescent images from live mice were acquired on a Leica SP8 using the Leica Application Suite (LAS) X software (version 3.7). Image stacks were processed, visualized, and analyzed using FIJI (Schindelin et al., Nat Meth 2012).

Light-sheet fluorescence microscopy
Hardware components for image acquisition (laser, camera, stage, filter wheel) were controlled by commercial software (IQ 2.9, Andor, Belfast, United Kingdom). Platelet (Alexa Fluor 750) and endothelium (Alexa Fluor 647) channels were deconvolved using the batch option in

Huygens Professional 20.04 (SVI, Netherlands). For the endothel channel automatic background subtraction was performed using the lowest value within a one-pixel radius, the signal to noise ratio was determined to 10 and a maximum of 40 iterations were performed. For the platelet channel, automatic background subtraction was done using the “in/near object” option with a radius of 2 µm (2 pixel). The signal to noise ratio was set to 40 and a maximum of 60 iterations were performed. The PSF of our home-build LSFM setup was characterized as follows for the deconvolution: detection NA = 0.15, refractive index = 1.554, NA of the illumination lens = 0.03, fill factor for the illumination lens = 0.5, illumination from left and a Gaussiand v9.0.2 an beam profile with a width of 18 µm.
The deconvolved data was converted into the Imaris file format ((Bitplane, Oxford Instruments, UK, version 9.6) and binned 2x in xy-direction to achieve 1024x1024 pixel per slice.
The data from the autofluorescence channel collected after 488 nm excitation was binned 2x in xy-direction to end up with 1024x1024 pixel per slice and imported into Ilastik (Berg et al., Nat Meth 2019). Ilastik was trained to recognize two classes “background” and “autofluorescence”. The respective probabilities for both classes were rescaled from 0 – 1 to 0 – 10’000 and exported as 16-bit unit tiff images. These images were then converted into the Imaris file format.
The preprocessed files from the three fluorescence channels was combined into a single Imaris file and further analyzed using its surface segmentation tool. The autofluorescence was segmented using the “autofluorescence” probabilities with an intensity threshold of 5’000 (thus reflecting 50% probability). Smaller, unconnected areas were removed manually. Next, the endothelium channel was segmented using a sample-dependent intensity threshold between 200-400 and a size filter of 1’000 voxel. For both channel segmentations, the surface grain size was left at 5.2 µm. The total volume from the autofluorescence reflecting the imaged brain volume and the total volume of the endothel system were exported. Finally, the thrombi were segmented using a surface grain size of 10 µm, a sample-dependent intensity threshold between 100-350 and a volume size filter of 1’000 µm³. Thrombi that were not in contact with the segmented vasculature were excluded from analysis. For each of the remaining and detected thrombi its volume was exported and a size distribution, mean and standard deviation calculated.

Observation of mouse behavior:
Mice were recorded using a standard webcam and videos were generated using DaVince Resolve (v17.2) software

Statistical analyses
No statistical methods were used to predetermine sample size. Data were collected in Microsoft Excel and statistical analysis was performed using GraphPad Prism software version 7.03 and v9.0.2 (GraphPad Software).

For manuscripts utilizing custom algorithms or software that are central to the research but not yet described in published literature, software must be made available to editors and reviewers. We strongly encourage code deposition in a community repository (e.g. GitHub). See the Nature Portfolio guidelines for submitting code & software for further information.

## Data

Policy information about availability of data

All manuscripts must include a data availability statement. This statement should provide the following information, where applicable:

- Accession codes, unique identifiers, or web links for publicly available datasets
- A description of any restrictions on data availability
- For clinical datasets or third party data, please ensure that the statement adheres to our policy

The datasets generated and/or analyzed during the current study are available in the source data file.

# Field-specific reporting

Please select the one below that is the best fit for your research. If you are not sure, read the appropriate sections before making your selection.

☒ Life sciences ☐ Behavioural & social sciences ☐ Ecological, evolutionary & environmental sciences

For a reference copy of the document with all sections, see nature.com/documents/nr-reporting-summary-flat.pdf

# Life sciences study design

All studies must disclose on these points even when the disclosure is negative.

| | |
|---|---|
| Sample size | No statistical methods were used to predetermine sample size. Pilot experiments and previously published results were used to estimate the sample size, such that appropriate statistical tests could yield significant results. However, for all experiments at least 3 mice per group were included and all experiments were performed at least twice. |
| Data exclusions | No data were excluded. |
| Replication | All experiments presented were conducted with sufficient mouse numbers to ensure statistical significance could be reached. Biochemical or image based data were reproduced in multiple mice. All attempts of replicating data were successfull. Experiments were replicated at least once to ensure validity. |
| Randomization | For the treatment groups, mice were randomized cage-wise and experiments were performed in a blinded manner during experiments and outcome assessment. The distribution of the mice into different groups was randomized using research randomizer (randomizer.org). |
| Blinding | Experiments with different treatment groups were blinded. Likewise, image analysis was performed in a blinded manner using code labeling of cleared organs (LSFM) or sections. |

# Reporting for specific materials, systems and methods

We require information from authors about some types of materials, experimental systems and methods used in many studies. Here, indicate whether each material, system or method listed is relevant to your study. If you are not sure if a list item applies to your research, read the appropriate section before selecting a response.

## Materials & experimental systems

| n/a | Involved in the study |
|---|---|
| ☐ | ☒ Antibodies |
| ☒ | ☐ Eukaryotic cell lines |
| ☒ | ☐ Palaeontology and archaeology |
| ☐ | ☒ Animals and other organisms |
| ☒ | ☐ Human research participants |
| ☒ | ☐ Clinical data |
| ☒ | ☐ Dual use research of concern |

## Methods

| n/a | Involved in the study |
|---|---|
| ☒ | ☐ ChIP-seq |
| ☐ | ☒ Flow cytometry |
| ☒ | ☐ MRI-based neuroimaging |

## Antibodies

**Antibodies used**

Description of all antibodies (clone and supplier) used in the study are provided in the Methods section of the Supplementary Material.
Commercial antibodies:
Anti-pan-phosphotyrosine antibody: Merck/Millipore, cat. nr. 05-321, clone 4G10, dilution 1:1000
Anti-CD31: BioLegend, cat.nr. 102402, clone390, dilution 1:100
Platelet-depletion antibody: emfret analytics, cat.nr. R300, 2 µg/g

In-house generated and in-house purified antibodies:
Anti-CLEC-2: clone INU1, in vivo: fab and F(ab')2 0.5µg/g, IgG 0.75 µg/g: in vitro: 10 µg/ml (Ref. May et al., Blood, 2009; Lorenz et al., Blood, 2015)
Anti-GPIIb/IIIa: clone JON/A, in vivo: F(ab)2 2 µg/g, for flow cytometry PE-conjugated IgG was used in saturating amounts were used (Ref. Bergmeier et al., Cytometry, 2002)
Anti-GPIbα-fab: clone p0p/B, 2 µg/g (Ref. Massberg et al., J Exp Med 2003)
Anti-GPIb-FITC: clone p0p4, saturating amounts were used for flow cytometry (Ref. Nieswandt et al., Blood 2000)
Anti-GPIIb/IIIa-PE: clone JON6, saturating amounts were used for flow cytometry (Ref. Nieswandt et al., Blood 2000)
Anti-P-Selectin-FITC: clone WUG1.9, saturating amounts were used for flow cytometry (Ref. May et al., Blood 2009)
Anti-FcγR: clone 2.4G2, 10 µg/ml (Ref. Unkeless, J Exp Med, 1979)
Anti-GPIX-derivative: LSFM and IVM 0.6 µg/g, PET 5 µg, in vitro 5 µg/ml (Ref. Stegner et al., Nat Commun 2017)
Anti-CD105: clone MJ7/18, 0.4 µg/g (Ref. Zehentmeier et al., Eur J Immunol, 2014)

**Validation**

Validation of commercial antibodies was done on a regular quality control of each lot by the manufacturer. For the pan-phosphotyrosine antibody, quality is routinely evaluated on EGF-treated human A431 carcinoma cells. The anti-CD31 antibody was quality control tested by immunofluorescent staining with flow cytometry analysis. BioLegend further tests each lot of antibody to an internally established “gold standard” to maintain lot-to-lot consistency. They conduct wide-scale stability studies to guarantee an accurate shelf-life for their products.
In house generated antibodies were validated against a “gold standard” reference lot and tested against respective isotype controls. If available, references of the used in house generated antibodies are given above.

## Animals and other organisms

Policy information about studies involving animals; ARRIVE guidelines recommended for reporting animal research

**Laboratory animals**

All animals are described in the Methods section.
For all experiments, male and female mice aged 8-20 weeks were used. Details regarding the individual experiments are given in the source data file. C57Bl/6J mice were purchased form Charles River. Clec1bY7A/Y7A (Ref. Haining et al.,Blood, 2017), Sykfl/fl, PF4Cre (Ref. van Eeuwijk et al., Arterioscler Thromb Vasc Biol, 2016) and Unc13d-/- (Ref. Stegner et al., J Thromb Haemost, 2013) were described previously. Animal studies were approved by the local authorities (District of Lower Franconia and Tübingen, AZ 13/13, 14/13, 2-348)

**Wild animals**

This study did not involve wild animals.

**Field-collected samples**

This study did not involve samples collected from the field.

**Ethics oversight**

Animal studies were approved by the District of Lower Franconia as stated in the Supplementary Methods section.

Note that full information on the approval of the study protocol must also be provided in the manuscript.

## Flow Cytometry

### Plots

Confirm that:

☒ The axis labels state the marker and fluorochrome used (e.g. CD4-FITC).

☒ The axis scales are clearly visible. Include numbers along axes only for bottom left plot of group (a 'group' is an analysis of identical markers).

☐ All plots are contour plots with outliers or pseudocolor plots.

☐ A numerical value for number of cells or percentage (with statistics) is provided.

### Methodology

| | |
|---|---|
| Sample preparation | Please see Supplementary Materials.<br>Blood was drawn from the retro-orbital plexus in heparin, diluted with PBS, stained with saturating amounts of the indicated antibodies. |
| Instrument | Becton Dickinson FACSCalibur |
| Software | CellQuest Pro (v6.0) |
| Cell population abundance | Cell population abundance: For in vitro experiments, population abundance of platelets was approx. 50%, for flow cytometry using whole blood, platelet population abundance was around 5% (lower for thrombocytopenic mice). |
| Gating strategy | Platelets were gated based on FSC/SSC characteristics and the gating strategy is provided in extended figure 1. |

☒ Tick this box to confirm that a figure exemplifying the gating strategy is provided in the Supplementary Information.
