# Supplementary material for: Foudroyant cerebral venous (sinus) thrombosis triggered through CLEC-2 and GPIIb/IIIa dependent platelet activation
Source: Nat Cardiovasc Res. 2022 Feb 10;1(2):132–41. doi: 10.1038/s44161-021-00017-1 (PMC11358028; doi:10.1038/s44161-021-00017-1)

Fig 1c

Uncropped Western Blot depicted in Fig. 1c (representative of n=3 blots)

Red rectangle indicates the part shown in the figure

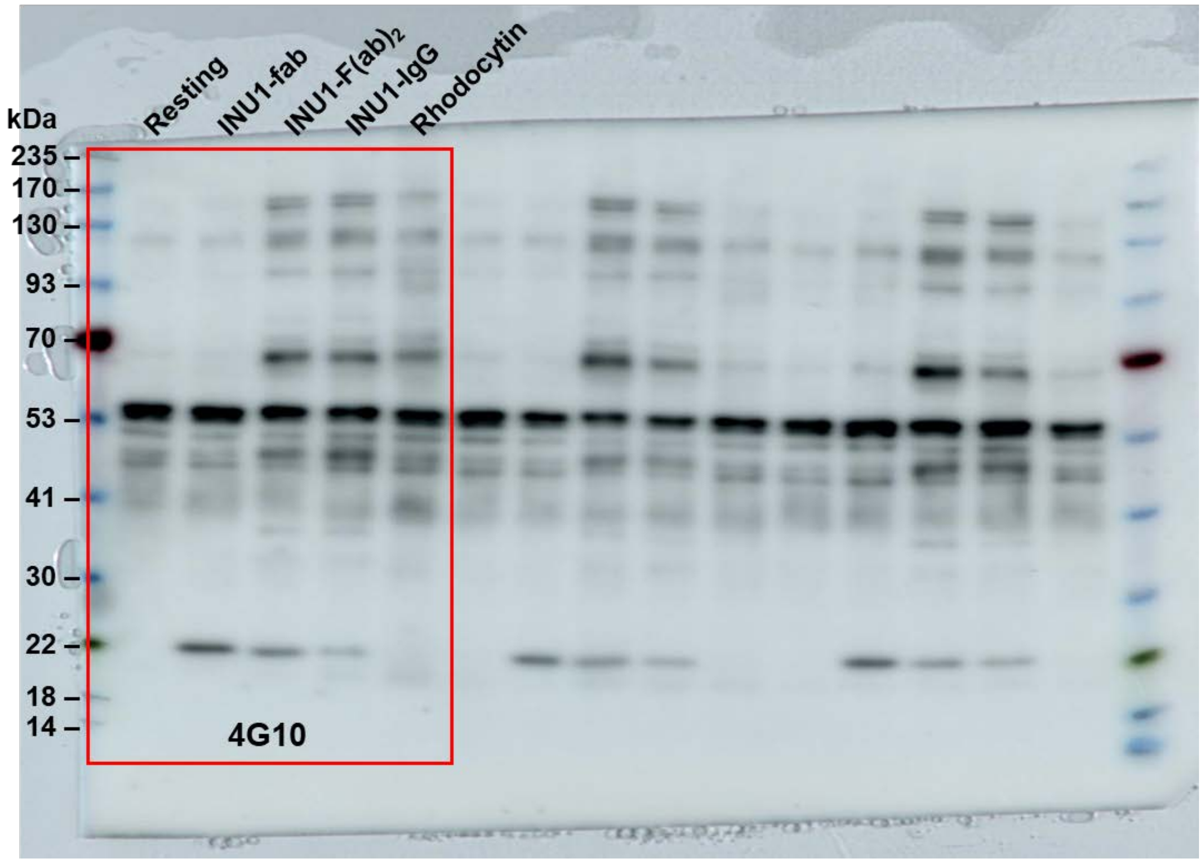

Supplement: Supplementary file 14 — uncropped gels of figure 1c [file 44161_2021_17_MOESM14_ESM.pdf]
